# PRMT inhibition induces a viral mimicry response in triple-negative breast cancer

**DOI:** 10.1038/s41589-022-01024-4

**Published:** 2022-05-16

**Authors:** Qin Wu, David Y. Nie, Wail Ba-alawi, YiShuai Ji, ZiWen Zhang, Jennifer Cruickshank, Jillian Haight, Felipe E. Ciamponi, Jocelyn Chen, Shili Duan, Yudao Shen, Jing Liu, Sajid A. Marhon, Parinaz Mehdipour, Magdalena M. Szewczyk, Nergiz Dogan-Artun, WenJun Chen, Lan Xin Zhang, Genevieve Deblois, Panagiotis Prinos, Katlin B. Massirer, Dalia Barsyte-Lovejoy, Jian Jin, Daniel D. De Carvalho, Benjamin Haibe-Kains, XiaoJia Wang, David W. Cescon, Mathieu Lupien, Cheryl H. Arrowsmith

**Affiliations:** 1 The Cancer Hospital of the University of Chinese Academy of Sciences (Zhejiang Cancer Hospital), Institute of Basic Medicine and Cancer (IBMC), Chinese Academy of Sciences, Hangzhou, China; 2 Structural Genomics Consortium, University of Toronto, Toronto, Ontario Canada; 3 Department of Medical Biophysics, University of Toronto, Toronto, Ontario Canada; 4 Princess Margaret Cancer Centre, University Health Network, Toronto, Ontario Canada; 5 School of Pharmaceutical Science and Technology, Tianjin University, Tianjin, China; 6 Molecular Biology and Genetic Engineering Center (CBMEG), Medicinal Chemistry Center (CQMED), Structural Genomics Consortium (SGC-UNICAMP), University of Campinas-UNICAMP, Campinas, Brazil; 7 Departments of Pharmacological Sciences and Oncological Sciences, Mount Sinai Center for Therapeutics Discovery, Tisch Cancer Institute, Icahn School of Medicine at Mount Sinai, New York, NY USA; 8 Ludwig Institute for Cancer Research, University of Oxford, Oxford, UK; 9 Institute for Research in Immunology and Cancer (IRIC), University of Montréal, Montréal, Quebec Canada; 10 Faculty of Pharmacy, University of Montreal, Montreal, Quebec Canada; 11 Department of Pharmacology and Toxicology, University of Toronto, Toronto, Ontario Canada; 12 Department of Computer Science, University of Toronto, Toronto, Ontario Canada; 13 Ontario Institute for Cancer Research, Toronto, Ontario Canada; 14 Vector Institute, Toronto, Ontario Canada

**Keywords:** Cancer therapy, Chemical genetics, RNA splicing, Screening

## Abstract

Triple-negative breast cancer (TNBC) is the most aggressive breast cancer subtype with the worst prognosis and few effective therapies. Here we identified MS023, an inhibitor of type I protein arginine methyltransferases (PRMTs), which has antitumor growth activity in TNBC. Pathway analysis of TNBC cell lines indicates that the activation of interferon responses before and after MS023 treatment is a functional biomarker and determinant of response, and these observations extend to a panel of human-derived organoids. Inhibition of type I PRMT triggers an interferon response through the antiviral defense pathway with the induction of double-stranded RNA, which is derived, at least in part, from inverted repeat Alu elements. Together, our results represent a shift in understanding the antitumor mechanism of type I PRMT inhibitors and provide a rationale and biomarker approach for the clinical development of type I PRMT inhibitors.

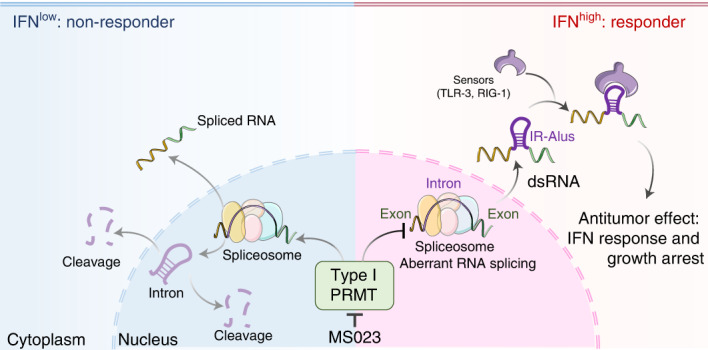

## Abstract (web summary)

Type I PRMT inhibition elicits potent antitumor activity associated with increased interferon response and intron-retained dsRNA accumulation, suggesting its potential combination with immune checkpoint inhibitors for cancer treatment.

## Main

Breast cancer is the world’s most commonly diagnosed cancer^1^. Triple-negative breast cancer (TNBC) is a heterogeneous breast cancer subtype lacking the expression of estrogen receptor, progesterone receptor and human epidermal growth factor receptor 2 (ref. ^2^). Although TNBC accounts for 15–20% of breast cancer cases, it leads to 25% of deaths^2^. Individuals with TNBC experience worse prognosis and overall survival rates than other subtypes due to both higher rates of recurrence (over 30%) and shorter survival following metastatic recurrence^3^. Due to the lack of targetable receptors that define other subtypes, options for TNBC treatment are limited, with chemotherapy representing the mainstay of current treatment. Despite some recent progress, new and effective targeted therapies for TNBC are urgently needed.

Epigenetic mechanism changes, such as DNA hypermethylation and chromatin modulator alteration, can disrupt regular development and lead to cancer^4^. Such evidence, coupled with recent progress in the development of drug-like small molecules capable of modulating epigenetic regulation, has fueled interest in exploiting the therapeutic potential of epigenetic targets^5^. Targeting epigenetic regulators, such as histone deacetylase inhibitors, BET bromodomain inhibitors and protein arginine methyltransferase 5 (PRMT5) inhibitors, has shown antitumor effects in TNBC and other cancers^6–9^. Despite these advances, the systematic understanding of epigenetic vulnerabilities in TNBC remains unclear.

To better understand how epigenetic regulators contribute to TNBC development and survival, we screened a collection of TNBC cell lines with a library of validated chemical probes that selectively target specific epigenetic regulators. Our screen revealed several previously reported epigenetic targets for TNBC, including enhancer of zeste homolog 2 (EZH2), BET bromodomains and PRMT5 (refs. ^6,7,10^). In addition to these, our results also revealed that type I PRMTs, the enzymes that catalyze asymmetric ω-*N*^G^,*N*^G^-dimethylarginine (ADMA) of histones and non-histone proteins, are important for TNBC growth. Among type I PRMTs, PRMT1 catalyzes around 85% of asymmetric dimethylation of proteins^11^. PRMT1-mediated arginine methylation is required for multiple cellular processes, including transcription, RNA splicing, cell communication and DNA repair^12^. mRNA splicing proteins have been identified as PRMT1 substrates, thus suggesting a role of PRMT1-dependent methylation in the regulation of RNA splicing^13^. Increasing evidence also demonstrates the functional roles of PRMT1 in DNA damage response by directly methylating DNA repair proteins, such as BRCA1, MRE11 and 53BP1 (refs. ^14,15^). Although cancer-associated mutations in PRMT1 are rare, its aberrant expression often correlates with poor outcome in various cancer types, including breast cancer^16^. Hence, the oncogenic potential of PRMT1 has been recognized, and it has recently received considerable attention as a potential therapeutic target in cancer^17^. Notably, GSK3368715, a type I PRMT inhibitor, has entered human clinical trials (NCT03666988), potentially opening up new avenues for the treatment of solid and hematological malignancies^17^. However, little is known about the therapeutic potential of type I PRMT inhibition in TNBC, and reliable biomarkers to identify tumors susceptible to type I PRMT inhibition are not known.

In this study, we developed a chemical screening approach to systematically investigate the epigenetic dependencies of TNBC and identified a specific vulnerability to the inhibition of type I PRMTs. We then examined the molecular factors underlying cell line-specific dependencies and validated our findings in human-derived models. Our data show that type I PRMT inhibition alters mRNA splicing, which leads to the expression of Alu sequences that can form cytosolic double-stranded RNA (dsRNA). This in turn triggers an antiviral interferon (IFN)-mediated response that pushes TNBC cells that are already stressed due to the elevated preexisting IFN response signature over a threshold to induce cell death.

## Results

### Type I PRMTs are key mediators of TNBC growth

**Fig. 1:**
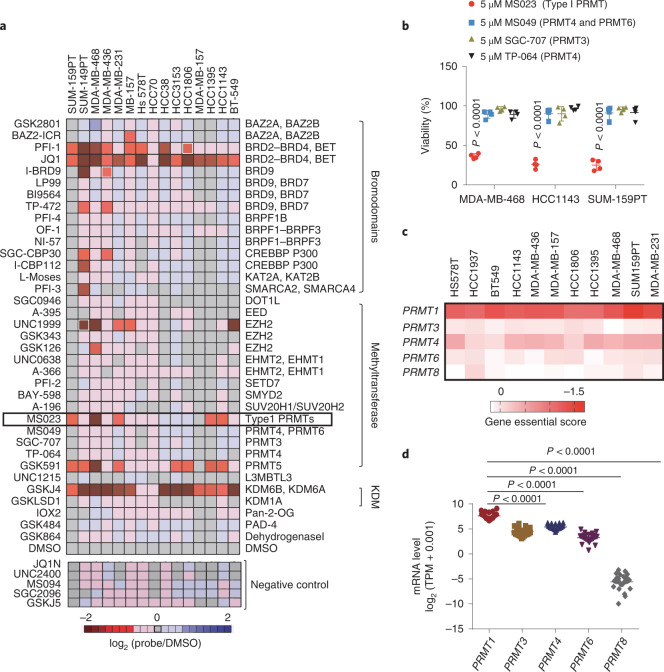
Chemical screen of 36 epigenetic probes identifies type I PRMTs as therapeutic targets in TNBC. **a**, Heat map showing the average cell proliferation values of the indicated epigenetic chemical probes at 6 d in 15 TNBC cell lines (data are shown as mean ± s.d. of *n* = 4); KDM, lysine demethylase. **b**, Viable cell counts of three TNBC cell lines treated with the indicated chemical probes for 7 d (data are shown as mean ± s.d. of *n* = 4); data were analyzed by one-way analysis of variance (ANOVA) with Dunnett’s test for multiple comparisons. **c**, Essential score of type I PRMTs across TNBC cell lines from the Cancer Dependency Map dataset (https://depmap.org/portal/). **d**, Type I PRMT mRNA expression in TNBC cell lines (*n* = 28 TNBC cell lines per group, each dot as an individual line); data were analyzed by one-way ANOVA with Dunnett’s test for multiple comparisons; TPM, transcripts per million. Source data

**Extended Data Fig. 1:**
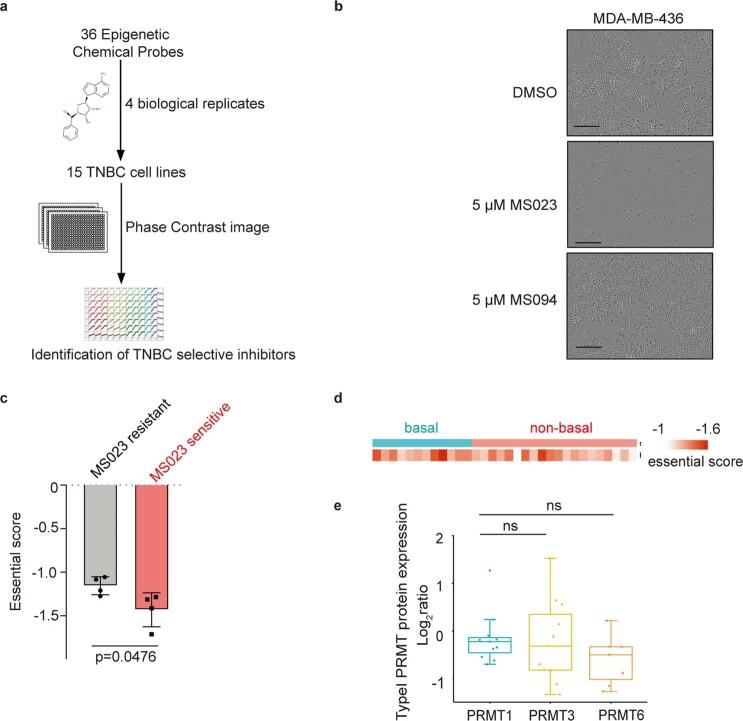
Dependency and expression of PRMTs across TNBC cell lines. **a**. Flow chart of the epigenetic-focused chemical screen: 36 epigenetic probes at 5 μM were tested in the indicated cell lines and images were recorded by an Incucyte Zoom System in order to calculate cell proliferation rate. **b**. A representative experiment showing effects of *in vitro* treatment of MDA-MB-436 cells with indicated compound. Images were captured by an Incucyte Zoom. Scale bar represents 300 µM (representative n = 4 independent experiments). c. Essential score of PRMT1 in MS023 sensitive and resistant cell lines (mean ± s.d., n = 4 independent TNBC cell line per group, Student’s two-tailed t-test). d. Essential score of PRMT1 in breast cancer cell lines (n = 32 breast cancer cell lines were tested). e. Protein expression of Type I PRMTs including PRMT1, PRMT3 and PRMT8 in Cancer Dependency Map dataset (https://depmap.org/portal/) datasets. Protein expression is reported as log_2_ ratio. In the boxplots, the centre lines mark the median, the box limits indicate the 25th and 75th percentiles, and the whiskers extend to 1.5× the interquartile range from the 25th and 75th percentiles (n = 11 TNBC cell line per group, unpaired two-tailed Student’s t-test). Source data

We first performed a cell-based chemical screen using a collection of 36 epigenetic chemical probes from the Structural Genomics Consortium (SGC) collection (https://www.thesgc.org/chemical-probes) to identify epigenetic regulators of TNBC cell growth^9,18^ (Supplementary Table 1). This chemical probe library includes tool compounds that selectively target key epigenetic regulatory proteins, including several epigenetic targets for which there are drugs currently in preclinical or clinical development. Each compound was evaluated in 15 TNBC cell lines, and cell confluency was assessed over 6 d using a live-cell imaging platform (Extended Data Fig. 1a). The primary screen identified six inhibitors that substantially reduced cell proliferation by more than 50% in at least one-third of the TNBC cell lines screened (Fig. 1a). BET bromodomain inhibitors (JQ1 and PFI-1) showed sensitivity across almost all the TNBC cell lines, as previously reported^6^. H3K27me3 methyltransferase and demethylase inhibitors (UNC1999 and GSKJ4) as well as a PRMT5 inhibitor (GSK591) also had antiproliferative effects on a wide variety of cancer cells, including TNBC^7,19,20^. Interestingly, a selective and cell-active inhibitor of type I PRMTs, MS023, showed growth-inhibitory properties across many TNBC cell lines, whereas its chemically similar but inactive control compound MS094 showed no effect (Fig. 1a and Extended Data Fig. 1b)^21^. Because type I PRMT inhibitors have potential broad applicability in diverse human cancers^13^ with at least one drug currently in clinical trials (NCT03666988), we sought to further characterize the action of type I PRMT inhibition in TNBC.

### PRMT1 is a major target among type I PRMTs in TNBC

Given the potency of MS023 against all type I PRMT members, including PRMT1, PRMT3, PRMT4, PRMT6 and PRMT8, we sought to understand whether a specific PRMT is the dominant target in TNBC. As illustrated in the screening heat map of cell confluence (Fig. 1a), inhibitors specifically targeting PRMT3 (SGC707) or PRMT4 (TP-064) and PRMT6 (MS049) showed no significant antiproliferative effect across the TNBC cell lines (Fig. 1a and Supplementary Table 2). Likewise, assessment of cell viability by CellTiter-Glo luminescent assay confirmed the profound inhibitory effect of MS023 on the representative TNBC cell lines, which was not observed with the other three inhibitors (Fig. 1b). These data rule out a major role for PRMT3, PRMT4 and PRMT6 in sustaining TNBC cell proliferation, individually.

Analysis of the public human Cancer Dependency Map dataset (https://depmap.org/portal/)^22,23^ revealed that among type I PRMTs, *PRMT1* knockout gives the highest essential gene score across a collection of TNBC cell lines, suggesting a critical role for PRMT1 in TNBC (Fig. 1c). Across these TNBC cells, the essentiality score for *PRMT1* was highest among those sensitive as opposed to resistant to MS023 treatment (Extended Data Fig. 1c). Nevertheless, some non-basal breast cancer cell lines are also dependent on PRMT1, indicating the potential of PRMT1 as a therapeutic target in these breast cancer types (Extended Data Fig. 1d). We then compared the expression level of type I PRMTs in our 15 TNBC cell lines and found that *PRMT1* mRNA was expressed at a higher level than other type I PRMTs (Fig. 1d and Extended Data Fig. 1e). Notably, *PRMT8* is barely expressed in TNBC, which is consistent with evidence of PRMT8 having brain-specific expression patterns (Fig. 1d)^24^. Taken together, we hypothesize that the observed efficacy of MS023 in TNBC is primarily due to inhibition of PRMT1 catalytic activity.

### PRMT1 inhibition suppresses tumor growth

**Fig. 2:**
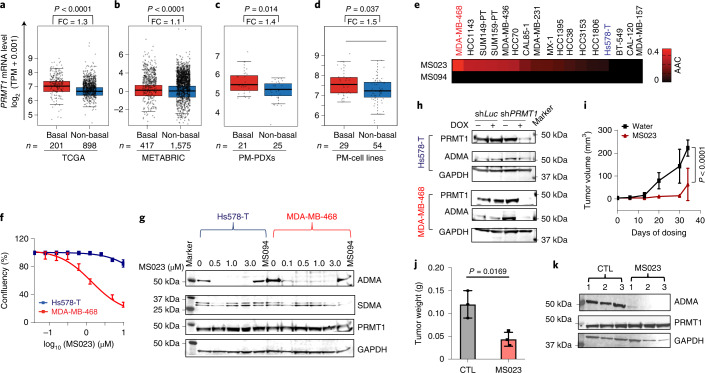
Type I PRMT inhibition suppresses tumor growth in a subset of TNBC. **a**–**d**, *PRMT1* gene expression in TCGA breast cancer datasets (**a**), METABRIC breast cancer datasets (**b**), Princess Margaret Hospital PDX datasets (PM-PDXs; **c**) and Princess Margaret Hospital cell line datasets (PM-cell lines; **d**). According to PAM50 classification, the cohorts were designated as basal and non-basal subtypes. Gene expression is reported as log_2_ (TPM + 0.001). In the box plots, the center lines mark the median, the box limits indicate the 25th and 75th percentiles, and the whiskers extend to 1.5× the interquartile range from the 25th and 75th percentiles. The numbers of individuals (*n*) per group are indicated, and the fold change (FC) values are as labeled. Data were analyzed by unpaired two-tailed Student’s *t*-test. **e**, Heat map of responsiveness to MS023 in the indicated cell lines. AAC was calculated from dose–response assays across 17 TNBC cell lines. Data are normalized to DMSO. A higher AAC indicates greater sensitivity. Colored cell lines are studied in more detail in this paper. Data are shown as mean ± s.d.; *n* = 4. **f**, Growth curves of Hs578-T and MDA-MB-468 cells treated with MS023 for 5 d. Data are shown as mean ± s.d.; *n* = 4. **g**, Immunoblots of MDA-MD-468 and Hs578-T cells following 5 d of treatment with the indicated doses of MS023 and the negative control MS094. Data are representative of *n* = 3 independent experiments. **h**, Immunoblots showing the doxycycline-inducible shRNA knockdown of *PRMT1* or luciferase control in MDA-MB-468 and Hs578-T cells. Data are representative of *n* = 3 independent experiments; SDMA, symmetric dimethylarginine; *Luc*, luciferase. **i**, Individual tumor growth of the MDA-MB-468 xenograft model with once-daily administration of 60 mg kg^–1^ MS023 when tumors reach 2 mm in diameter. Data are shown as mean ± s.d.; *n* = 3. Data were analyzed by two-way ANOVA with Dunnett’s test for multiple comparisons. **j**, Tumor weight was measured as a surrogate for tumor burden from the control (CTL) and MS023-treated mice. Data are shown as mean ± s.d. (*n* = 3) and were analyzed by one-way ANOVA with Dunnett’s test. **k**, Immunoblot of tumor tissue from mice treated with control or MS023 at the experimental endpoint. Data are representative of *n* = 3 independent technical experiments. Source data

Through reviewing clinical data from two large The Cancer Genome Atlas (TCGA) and Molecular Taxonomy of Breast Cancer International Consortium (METABRIC) cohorts, we further evaluated TNBC’s dependency on PRMT1 and compared the expression levels of PRMT1 in TNBC to other subtypes^25,26^. The analysis demonstrated that *PRMT1* mRNA expression is significantly higher in the basal-like subtype^13^ than in other breast tumor subtypes (Fig. 2a,b). This finding is further confirmed in a small cohort of independent breast cancer human-derived xenograft (PDX) and cell line^27^ cohorts (Fig. 2c,d).

**Extended Data Fig. 2:**
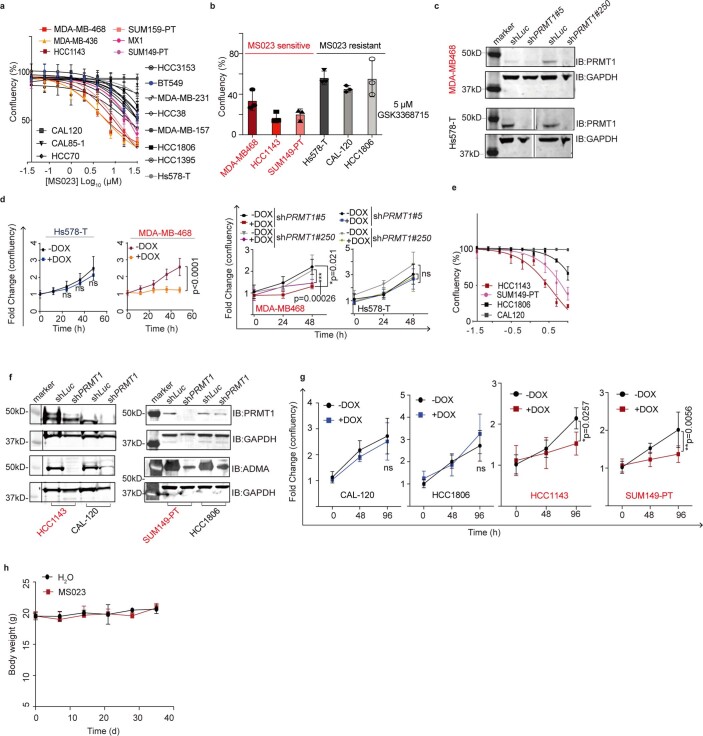
PRMT1 inhibition suppresses cell growth in a subset of TNBC cell lines. **a**. Growth curves of 17 TNBC cell lines with MS023 at indicated concentrations for 5 days treatment (means±s.d., n = 4). **b**. Cell confluency of TNBC cells with 5 μM GSK3368715 treatment for 5 days (means±s.d., n = 3). **c**. Immunoblots showing two more shRNA knockdown of PRMT1 in both MDA-MB-468 and Hs578-T cells (representative of n = 3 independent experiments). **d**. Normalized cell confluence of PRMT1 knockdown for the indicated time post doxycycline induction (means±s.d., n = 4, two-way ANOVA with Dunnett’s test for multiple comparisons, ns: not significant). **e**. Growth curves of 4 TNBC cell lines with MS023 at indicated concentrations for 5 days treatment (means±s.d., n = 4). **f**. Representative immunoblots showing shRNA knockdown of PRMT1 in TNBC cell lines (red: MS023 sensitive; black: MS023 resistant) (representative of n = 3 independent experiments). **g**. Normalized cell confluence of PRMT1 knockdown for the indicated time post doxycycline induction (means±s.d., n = 4, two-way ANOVA with Dunnett’s test for multiple comparisons, ns: not significant). **h**. Body weights measurement of individual mouse over the course of treatment with control or MS023 (means±s.d., n = 3). Source data

Next, we performed dose–response assays with nine concentrations (ranging from 40 nM to 10 µM) of MS023 across 17 TNBC cell lines and assayed the response using a live-cell imaging platform to monitor cell confluence over time (Extended Data Fig. 2a). We calculated the area above the curve (AAC) to capture the efficacy and potency of the inhibitor, with a higher AAC value indicating greater sensitivity to MS023 treatment (Fig. 2e). These highly reproducible responses identified both sensitive (for example, MDA-MB-468) and resistant (for example, Hs578-T) cell lines, suggesting that MS023 is not universally cytostatic and that specific determinants of sensitivity exist within TNBC models. A representative experiment shows the effects of MS023 on TNBC cell growth in both sensitive and resistant cell lines over 5 d (Fig. 2f). A similar differential inhibitory pattern was observed with the clinical inhibitor GSK3368715 in MS023-sensitive and MS023-resistant cells (Extended Data Fig. 2b).

Given that PRMT1 is the most abundant type 1 PRMT enzyme, we investigated whether the antiproliferative effect of MS023 coincides with a reduction of the ADMA mark. To address this, we measured ADMA levels in a sensitive cell line (MDA-MB-468) following MS023 treatment for 5 d (Fig. 2g). We observed decreased ADMA levels in a dose-dependent manner over the dose range from 0.1 µM to 3 µM, which had a significant suppressive effect on proliferation of the MDA-MB-468 cell line. The observed PRMT1-dependent effect of MS023 inhibition was not due to changes in PRMT1 levels, as its expression was not altered following treatment (Fig. 2g). No significant differences in symmetric dimethylarginine levels were observed between sensitive and resistant cells in response to type I PRMT inhibition, indicating that the resistance to MS023 is not due to symmetric dimethylarginine compensation (Fig. 2g). MS023 treatment also reduced the ADMA mark in the resistant cell line Hs578-T, ruling out the possibility that differential sensitivity was attributable to drug efflux pumps or other mechanisms that might simply prevent target modulation by MS023.

We then performed *PRMT1* genetic knockdown assays to confirm the on-target effect of type I PRMT inhibition. Knockdown of *PRMT1* using three different inducible short hairpin RNAs (shRNAs) reduced PRMT1 expression and ADMA marks in both cell lines (Fig. 2h and Extended Data Fig. 2c). As observed with MS023 treatment, *PRMT1* knockdown substantially suppressed cell growth in the sensitive cell lines (MDA-MB-468, HCC1143 and SUM149-PT) but not in the resistant cell lines (Hs578-T, CAL-120 and HCC1806; Extended Data Fig. 2d–g). Taken together, these data support that the antiproliferative effect of MS023 in a subset of TNBC cell lines is due to inhibition of PRMT1 activity.

To determine whether the in vitro effects observed above can translate to antitumor activity in vivo, we next evaluated the efficacy and tolerability of MS023 in vivo. Mice bearing MDA-MB-468 xenografts were treated with control (water) or MS023, respectively. MS023 was well tolerated, with no difference in body weight of animals treated with MS023 versus control-treated animals over 35 d (Extended Data Fig. 2h). Once-daily dosing of 60 mg kg^–1^ MS023 initiated in mice with palpable tumors significantly reduced tumor growth and final tumor weight (Fig. 2i,j)^28^. Notably, MS023 treatment reduced the ADMA mark of the xenograft tissue, suggesting that the reduction of tumor size coincides with disruption of the enzymatic function of PRMT1 (Fig. 2k). While this exploratory pilot assay requires further confirmation with more in vivo studies, our data collectively suggest that type I PRMT inhibition, primarily through PRMT1 inhibition, may be a promising therapeutic strategy for the treatment of a subset of TNBCs.

### Preexisting IFN signaling correlates with MS023 sensitivity

**Fig. 3:**
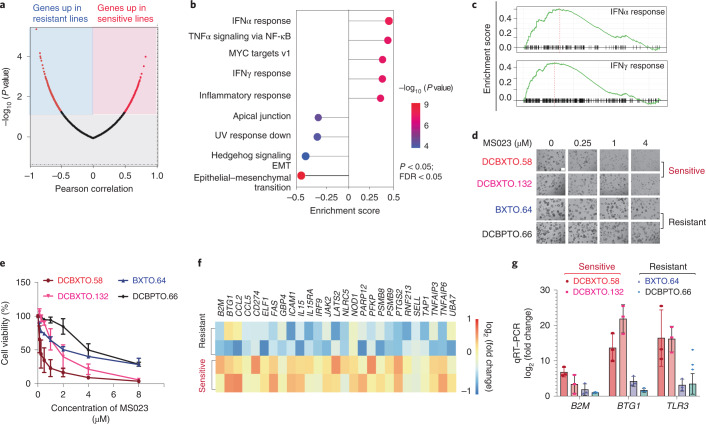
Increased IFN responses underlie the responsiveness to type I PRMT inhibition. **a**, Pearson correlation analysis of individual gene expression and MS023 activity. A Volcano plot of log_2_ fold change values for all genes significantly upregulated in sensitive lines (red, left) or in resistant lines (blue, right) is shown. Data were analyzed by unpaired two-tailed Student’s *t*-test for multiple comparisons. **b**, Top nine pathways significantly correlated with MS023 sensitivity. Data were analyzed by one-tailed Fisher’s exact test for multiple comparisons; FDR, false discovery rate; EMT, epithelial–mesenchymal transition. **c**, GSEA for gene sets associated with IFN responses enriched in sensitive TNBC lines. Data were analyzed by one-tailed Fisher’s exact test. **d**, Images of different organoid models with either DMSO or MS023 treatment; scale bar, 100 μm. The image shown is representative of *n* = 4 independent experiments. **e**, Differential response of organoids to MS023 treatment. Data are shown as mean ± s.d.; *n* = 2. **f**, Heat map showing the differential expression of IFN-responsive genes between sensitive and resistant models. **g**, qRT–PCR validation of the expression of the indicated genes in both MS023-sensitive and MS023-resistant organoid models. Data are shown as mean ± s.d.; *n* = 3. Source data

**Extended Data Fig. 3:**
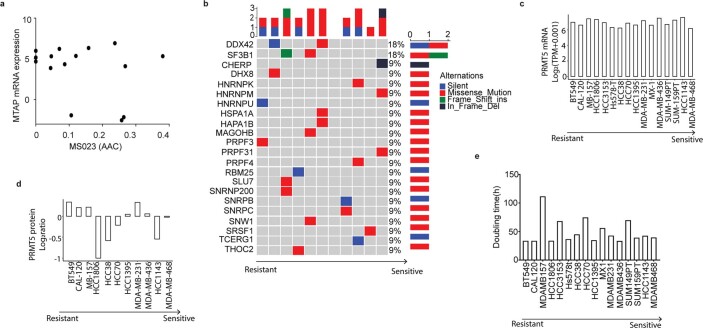
No significant identified molecular signature correlates with MS023 sensitivity in TNBC. **a**. Correlation of MTAP expression and MS023 sensitivity. **b**. Oncoplot showing the mutation status of genes involved in RNA splicing across TNBC cell lines. Color indicates different mutation type. Arrow indicates the sensitivity of cell lines to MS023 treatment. **c**. PRMT5 gene expression in the Princess Margaret Hospital cell line datasets (PM-Cell lines) datasets. Gene expression is reported as log_2_(TPM + 0.001). **d**. PRMT5 protein expression in TNBC cell lines. Arrow indicates the sensitivity of cell lines to MS023 treatment. **e**. Correlation of cell doubling time and MS023 sensitivity. Source data

Although PRMT1 has been implicated in multiple biological processes, such as transcriptional modulation, pre-mRNA splicing and receptor signaling^13^, the extent to which any of these pathways contributes to the PRMT1 dependency of TNBC tumor cells is not clear. Previous studies have identified the association between sensitivity of PRMT1 inhibition and methylthioadenosine phosphorylase (MTAP) level, although this correlation in breast cancer is not as strong as in lymphoma^17^. Our data show no correlation between the response to PRMT1 inhibition and MTAP deficiency (Extended Data Fig. 3a). A recent report showed that mutation of serine- and arginine-rich splicing factor 2 (SRSF2) sensitizes cells to type I PRMT inhibition in leukemia^28^. However, we found that SRSF status or other splicing factor mutations appear unrelated to PRMT1 sensitivity in TNBC (Extended Data Fig. 3b). Given the synergistic effect between PRMT1 and PRMT5 (ref. ^29^), we investigated PRMT5 expression and MS023 sensitivity. Neither the mRNA nor protein level of PRMT5 was predictive of MS023 response (Extended Data Fig. 3c,d). Having ruled out these candidates, we performed systematic global profiling of basal gene expression for our panel of 17 TNBC cell lines^27^ and calculated the Pearson correlation coefficients of MS023 activity (as measured by the AAC value of the MS023 dose–response curve in Fig. 2e) with individual gene expression levels (Fig. 3a). We then subjected these rank-ordered gene lists to gene set enrichment analysis (GSEA). The IFNα response pathway was the most enriched pathway, with several additional immune-related pathways, including IFNγ response and tumor necrosis factor-α signaling via NF-κB pathways, also among the most enriched in MS023-sensitive lines (Fig. 3b,c). Given the link between replication stress and IFN response, we calculated the doubling time of each TNBC cell line and found no correlation between the proliferation rate and MS023 sensitivity (Extended Data Fig. 3e). Altogether, our results suggest that a preexisting enhanced expression of genes involved in the IFN signaling pathway might predict the responsiveness to MS023.

To better address this hypothesis in more clinically relevant TNBC models, we tested whether the correlation between IFN signaling gene signatures and MS023 sensitivity was also evident across human-derived TNBC samples. Three-dimensional cancer organoid models are thought to better recapitulate clinical disease and epithelial heterogeneity in TNBC and are therefore regarded as a superior tool for the evaluation of drug responses^30^. Four different organoid models were treated with either DMSO or MS023 at dose ranges from 0 µM to 10 µM and assessed by microscopic evaluation and PrestoBlue staining. Representative images of these organoid models (Fig. 3d) and the quantified percent cell viability of each model and treatment conditions are shown (Fig. 3e). Using the transcriptomic data of tumors matching these organoid models, the differential expression of IFN response genes was studied between the MS023-sensitive and MS023-resistant organoid models (Fig. 3f). Consistently, organoids with higher basal IFN gene expression were more sensitive to MS023 treatment, while organoids with lower IFN gene expression were more resistant. Quantitative real-time PCR (qRT–PCR) of RNA extracted from organoids further confirmed the upregulation of type 1 helper T cell chemokines (for example, *CCL2* and *CCL5*) and antigen presentation genes (for example, *B2M* and *BTG1*) in sensitive models (Fig. 3g). Taken together, our findings across a range of human-derived models suggest that the preexisting levels of IFN response signatures are correlated with the degree of sensitivity to type I PRMT inhibition.

### Type I PRMT inhibition triggers IFN responses

**Fig. 4:**
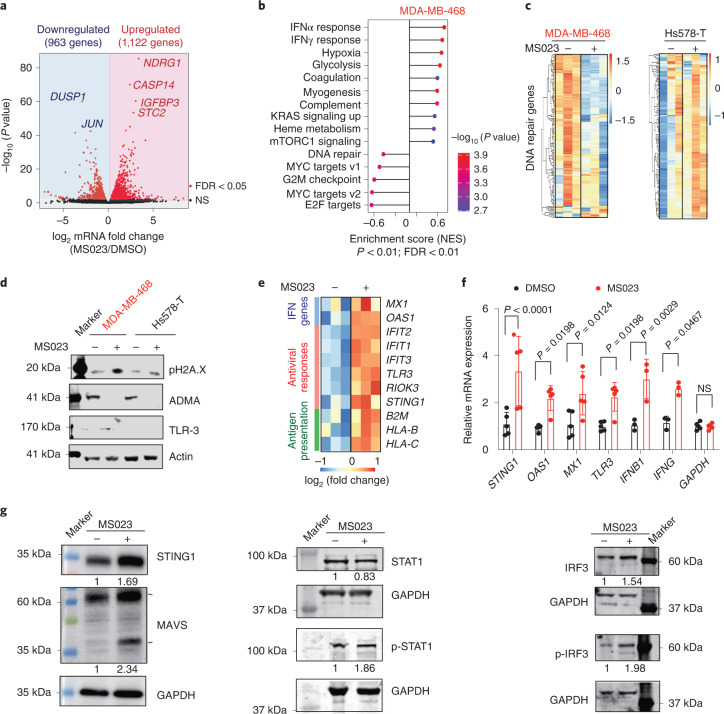
MS023 treatment triggers IFN responses. **a**, Volcano plot of log_2_ fold change for genes significantly upregulated (red, right) or downregulated (blue, left) following 5 d of MS023 treatment in the MDA-MB-468 cell line. Data were analyzed by unpaired two-tailed Student’s *t*-test; NS, not significant. **b**, GSEA of all ranked differentially expressed genes. Data were analyzed by one-tailed Fisher’s exact test; NES, normalized enrichment score. **c**, Heat map showing genes associated with DNA repair after MS023 treatment for 5 d in both MDA-MB-468 and Hs578-T cell lines. **d**, Immunoblots showing the expression level of pH2AX, a DNA damage marker, with either DMSO or MS023 treatment in both MDA-MB-468 and Hs578-T cell lines. Data are representative of *n* = 3 independent experiments. **e**, Heat map showing RNA-seq data for indicated genes in MDA-MB-468 cells with either DMSO or MS023 treatment for 5 d. **f**, Expression of indicated IFN-responsive genes in MDA-MB-468 cells with either DMSO or MS023 treatment for 5 d analyzed by qRT–PCR. Data are shown as mean ± s.d.; *n* = 5 for *STING1*, *OAS1*, *MX1*, *TLR3* and *GAPDH*; *n* = 3 for *IFNB1* and *IFNG*. Data were analyzed by two-way ANOVA with Dunnett’s test for multiple comparisons. **g**, Immunoblots of the indicated proteins in MDA-MB-468 cells after 5 d of MS023 treatment. Normalized band intensity is labeled. Data are representative of *n* = 3 independent experiments; p-STAT1, phospho-STAT1; p-IRF3, phospho-IRF3. Source data

**Extended Data Fig. 4:**
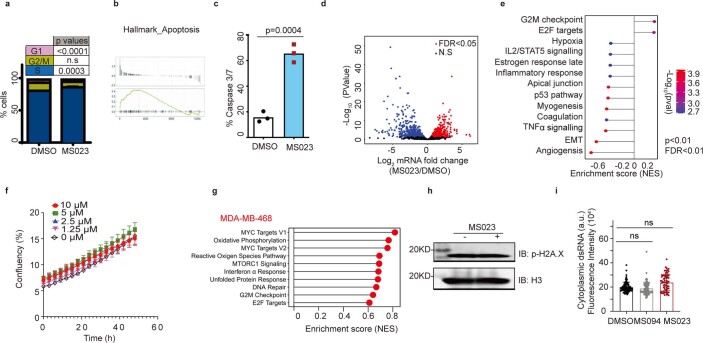
Type I PRMT inhibition stimulates interferon responses in a subset of TNBC cell lines. **a**. Flow cytometry cell cycle analysis for MDA-MB-468 cells cultured with or without MS023 for 5 days (means±s.d., n = 3, two-way ANOVA with Dunnett’s test for multiple comparisons, ns: not significant). **b**. GSEA of the apoptosis pathway in MDA-MB-468 cells after MS023 treatment for 5 days. (one-tailed Fisher’s exact test for multiple comparisons). **c**. Apoptotic cell counts of MS023 treated for 5 days by caspase 3/7 staining (means±s.d., n = 3, two-sided Student’s t test). **d**. Volcano plot of log_2_ fold change for genes significantly upregulated (red in the right panel) or downregulated (blue in left panel) upon MS023 treatment (n = 3) in Hs578-T cell line (unpaired two-tailed Student’s t-test). **e**. Gene set enrichment analysis (GSEA) of all ranked differential expressed genes in Hs578-T after 5 days MS023 treatment (one-tailed Fisher’s exact test for multiple comparisons). **f**. Growth curves of MDA-MB-468 cell with indicated concentration of MS023 for 2 days culture (means±s.d., n = 4). **g**. Top 10 gene set enrichment analysis (GSEA) of all ranked differential expressed genes in MDA-MB-468 after 2 days MS023 treatment. **h**. Representative immunoblots showing DNA damage after MS023 treatment for 2 days (one of three independent experiments are shown). **i**. Quantification of cytoplasmic dsRNA signal intensity in MDA-MB-468 cells with indicated compound treatment for 2 days (means±s.d., n = 3 independent biologically experiments of at least 86 cells per group, one-way ANOVA with Dunnett’s test for multiple comparisons). Source data

To better understand the potential mechanisms underlying the effects of type I PRMT inhibition in TNBC, we performed RNA sequencing (RNA-seq) on MDA-MB-468 cells following 5 d of treatment with MS023 and identified 2,085 genes as significantly differentially expressed (*P* < 0.05, false discovery rate < 0.05; Fig. 4a). Hallmark enrichment analysis revealed that E2F targets and G2M checkpoint pathways were downregulated after MS023 treatment, suggesting that type I PRMT inhibition affects the expression of genes that function in cell cycle regulation (Fig. 4b). In agreement, MS023 treatment leads to a modest but significant decrease in S phase and a concurrent increase in G1 phase (Extended Data Fig. 4a)^17^. Also consistent with the antitumor effect of MS023 observed in sensitive cell lines, GSEA revealed induction of the apoptosis pathway following MS023 treatment (Extended Data Fig. 4b). This was confirmed by the increased number of apoptotic cells after MS023 treatment (Extended Data Fig. 4c). In addition to the above noted effects, which likely reflect the downstream consequences of type I PRMT inhibition, a comparison of the differential gene expression patterns in both MDA-MB-468 (sensitive) and Hs578-T (resistant) lines showed that 5 d of treatment with MS023 decreased the expression of DNA repair genes in the sensitive line but not in the resistant line (Fig. 4c and Extended Data Fig. 4d,e). Western blotting of MDA-MB-468 cells revealed that the phosphorylation of histone H2AX at Ser 139 (pH2AX), a marker of DNA damage, was enhanced after MS023 treatment (Fig. 4d). To rule out potential non-specific effects induced by compound toxicity, we again treated MDA-MB-468 cells with MS023 but only for 2 d. No significant cell growth suppression (Extended Data Fig. 4f), E2F/G2M pathway downregulation (Extended Data Fig. 4g), DNA damage or dsRNA accumulation (Extended Data Fig. 4h,i) was observed at this earlier time point, consistent with a growth-suppressive mechanism other than acute toxicity.

GSEA further suggested an innate immune response mechanism, which could contribute to the antiproliferative phenotype. Upregulation of the IFNα and IFNγ innate immune response pathways were prominent GSEA signatures triggered by type I PRMT inhibition in MDA-MB-468 cells (Fig. 4b,c). Among the top upregulated genes after MS023 treatment, we observed increased expression of genes such as IFN-responsive genes of IFNβ and IFNγ and dsRNA-sensing pathway genes of STING1 and TLR-3. These markers of an antiviral stress response were observed both in RNA-seq and subsequent qRT–PCR validation (Fig. 4e,f). Similarly, western blotting identified activation of the dsRNA-sensing pathway, as reflected by increased STAT1 and IRF3 phosphorylation as well as the expression of STING1 and MAVS after MS023 treatment in the sensitive cell line MDA-MB-468 (Fig. 4g). Together, these results identified a stress response to elevated dsRNA as a potential result of type I PRMT inhibition, which could underlie its antiproliferative and cytotoxic effects.

### Type I PRMT inhibition induces dsRNA accumulation

**Fig. 5:**
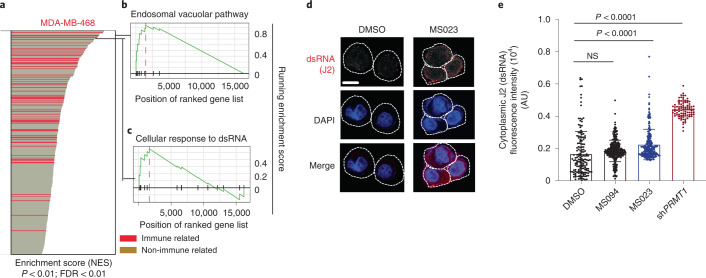
Type I PRMT inhibition induces cytoplasmic dsRNA formation due to increased intron retention. **a**–**c**, Type I PRMT inhibition leads to activation of immune signatures in MS023-sensitive cells. **a**, Bar plot of upregulated gene sets enriched in the MDA-MB-468 cell line after MS023 treatment. Gene sets associated with immune response are red. Data were analyzed by one-tailed Fisher’s exact test. **b**, GSEA for gene sets associated with the dsRNA-sensing endosomal vacuolar pathway. Data were analyzed by one-tailed Fisher’s exact test. **c**, GSEA for gene sets associated with cellular response to dsRNA. Data were analyzed by one-tailed Fisher’s exact test. **d**, Cellular dsRNA was evaluated by anti-dsRNA (J2) immunofluorescence; scale bar, 10 μm. The images shown are representative of *n* = 3 independent experiments. **e**, Quantification of cytoplasmic dsRNA signal intensity; AU, arbitrary units. Data are shown as mean ± s.d.; *n* = 3 independent biological experiments of at least 85 cells per group analyzed. Data were analyzed by one-way ANOVA with Dunnett’s test for multiple comparisons. Source data

**Extended Data Fig. 5:**
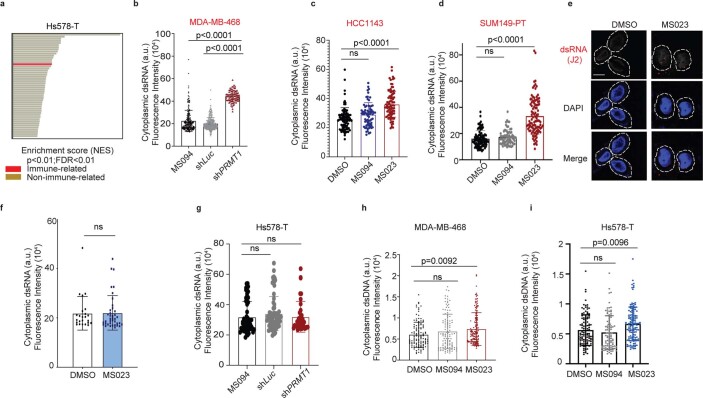
MS023 triggers interferon response through dsRNA accumulation. **a**. Upregulated gene sets enriched in Hs578-T cell after MS023 treatment for 5 days. Gene sets associated with immune response are red (one-tailed Fisher’s exact test). **b**. Quantification of cytoplasmic dsRNA signal intensity in MDA-MB-468 cells with indicated compound or shPRMT1 for 5 days (means±s.d., n = 3 independent biologically experiments of at least 91 cells per group analyzed). **c**. Quantification of cytoplasmic dsRNA signal intensity in HCC1143 cells with indicated compound treatment for 5 days (means±s.d., n = 3 independent biologically experiments of at least 70 cells per group analyzed). **d**. Quantification of cytoplasmic dsRNA signal intensity in SUM149-PT cells with indicated compound treatment for 5 days (means±s.d., n = 3 independent biologically experiments of at least 70 cells per group analyzed). **e**. Images of cellular dsRNA staining in Hs578-T cell after MS023 treatment for 5 days. Scale bar represents 10 μm (Representative images of 3 independent experiments are shown). **f**. Quantification of cytoplasmic dsRNA signal intensity in Hs578-T cell after MS023 treatment for 5 days (means±s.d., n = 3 of at least 40 cells per group analyzed, one-way ANOVA with Dunnett’s test). **g**. Quantification of cytoplasmic dsRNA signal intensity in Hs578-T cells with indicated compound or shPRMT1 for 5 days (means±s.d., n = 3 independent biologically experiments of at least 45 cells per group analyzed). **h**. Quantification of cytoplasmic dsDNA signal intensity in MDA-MB-468 cells with indicated compound treatment for 5 days (means±s.d., n = 3 independent biologically experiments of at least 120 cells per group analyzed). **i**. Quantification of cytoplasmic dsDNA signal intensity in Hs578-T cells with indicated compound treatment for 5 days (means±s.d., n = 3 independent biologically experiments of at least 43 cells per group analyzed). For panels **b-d**, and **g-i**, *P* values were calculated using one-way ANOVA with Dunnett’s test for multiple comparisons. Source data

To investigate whether and how MS023 treatment may trigger a viral mimicry response in TNBCs, gene ontology (GO) analysis was performed in both MDA-MB-468 (sensitive) and Hs578-T (resistant) cell lines. MS023 induced immune-related responses in sensitive lines to a much greater extent than the resistant line (Fig. 5a and Extended Data Fig. 5a). Gene sets including cellular response to dsRNA and dsRNA-sensing signaling (endosomal vacuolar pathway) were two of the top significant positively enriched pathways (Fig. 5b,c). To further validate dsRNA formation, immunofluorescence staining with a J2 antibody (a gold standard for dsRNA detection^31^) identified that either genetic or pharmacological inhibition of PRMT1 induced a robust increase of cytoplasmic dsRNA in MS023-sensitive cell lines (Fig. 5d,e and Extended Data Fig. 5b–d) but not in the MS023-resistant lines (Extended Data Fig. 5e–g). We also compared dsDNA formation in both MS023-sensitive (MDA-MB-468) and MS023-resistant (Hs578-T) cell lines and found that dsDNA is accumulated to a similar extent in both cell lines after MS023 treatment, ruling out a major role for dsDNA in differential IFN activation in MDA-MB-468 cells (Extended Data Fig. 5h,i).

**Fig. 6:**
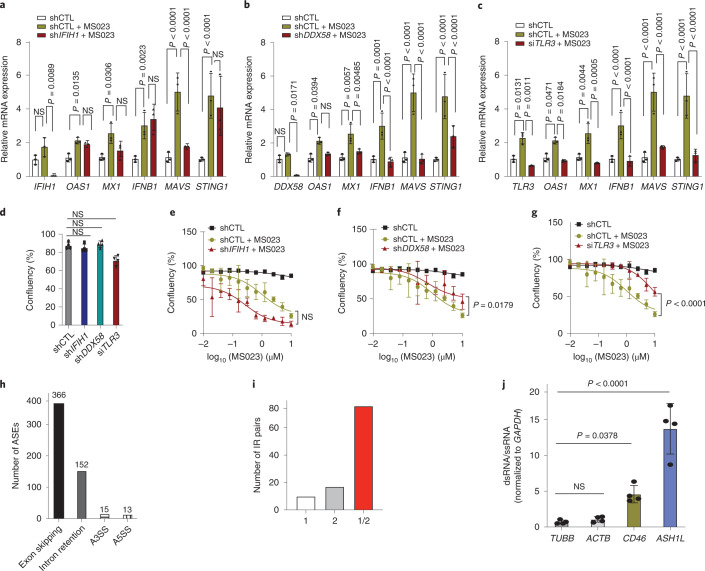
MS023 activates dsRNA sensors to induce IFN signaling. **a**, The expression of *IFIH1* and indicated IFN-responsive genes in scramble-treated (shCTL), MS023-treated and *IFIH1*-knockdown/MS023-treated MDA-MB-468 cells analyzed by qRT–PCR. **b**, The expression of *DDX58* and indicated IFN-responsive genes in scramble-treated, MS023-treated and *DDX58*-knockdown/MS023-treated MDA-MB-468 cells analyzed by qRT–PCR. **c**, The expression of *TLR3* and indicated IFN-responsive genes in scramble-treated, MS023-treated and *TLR3*-knockdown/MS023-treated MDA-MB-468 cells analyzed by qRT–PCR. Data in **a**–**c** are shown as mean ± s.d. (*n* = 3) and were analyzed by one-way ANOVA with Dunnett’s test for multiple comparisons. **d**, Cell confluence of scramble-treated, *IFIH1*-knockdown, *DDX58*-knockdown and *TLR3*-knockdown MDA-MB-468 cells. Data are shown as mean ± s.d. (*n* = 4) and were analyzed by one-way ANOVA with Dunnett’s test for multiple comparisons. **e**, Cell confluence of scramble-treated, MS023-treated and *IFIH1*-knockdown/MS023-treated MDA-MB-468 cells. **f**, Cell confluence of scramble-treated, MS023-treated and *DDX58*-knockdown/MS023-treated MDA-MB-468 cells. **g**, Cell confluence of scramble-treated, MS023-treated and *TLR3*-knockdown/MS023-treated MDA-MB-468 cells. Data in **e**–**g** are shown as mean ± s.d. (*n* = 4) and were analyzed by two-way ANOVA with Dunnett’s test for multiple comparisons. **h**, Bar plot showing the number of ASEs belonging to each of the main alternative splicing categories. **i**, Bar plot showing the count of IR pairs in which the intron intersects with only the first Alu in the pair (1; white), with only the second Alu in the pair (2; gray) or with the two Alus in the pair (1/2; red). **j**, qRT–PCR analysis of the indicated genes after J2 immunoprecipitation. Data are shown as mean ± s.d. (*n* = 4) and were analyzed by one-way ANOVA with Dunnett’s test for multiple comparisons; ssRNA, single-stranded RNA. Source data

MDA5 (IFIH1), RIG-I (DDX58) and TLR-3 are receptors that bind to intracellular dsRNA^32^. To understand the individual contributions of these dsRNA sensors in IFN activation, we reduced their expression in MDA-MB-468 cells with validated shRNAs or short interfering RNA. Knockdown of *DDX58* or *TLR-3* decreased MS023-mediated upregulation of IFN genes *IFNB1*, *MX1* and *OAS1* and rescued the MS023 growth inhibitory effect (Fig. 6a–g). However, *IFIH1* knockdown had no significant effect on MS023-induced IFN gene expression and cell growth (Fig. 6a,e). These results suggest that RIG-I (DDX58) and TLR-3, which preferentially recognize distinct pools of dsRNA^32^, are responsible for IFN activation in response to MS023 treatment.

**Extended Data Fig. 6:**
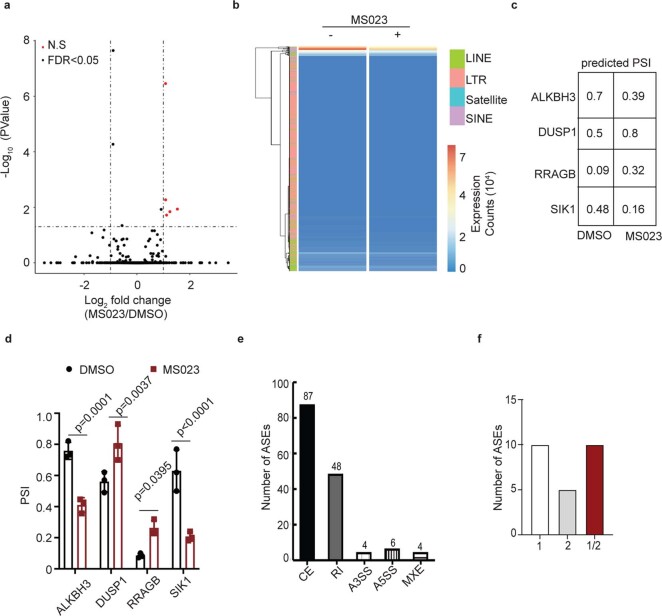
Retained introns contribute to dsRNA accumulation in MS023 sensitive cells. **a**. Volcano plot depicting expression changes of transposable elements in MDA-MB-468 cells after MS023 treatment for 5 days (unpaired two-tailed Student’s t-test). **b**. Heatmap depicting expression changes of transposable elements in MDA-MB-468 cells after MS023 treatment for 5 days (unpaired two-tailed Student’s t-test). **c**. Predicted PSI (percent spliced in) of indicated genes in MDA-MB-468 cells with indicated treatment for 5 days. **d**. PSI values derived from RT-PCR analysis for indicated genes from MDA-MB-468 cells after MS023 treatment for 5 days (means±s.d., n = 3, one-way ANOVA with Dunnett’s test for multiple comparisons). **e**. Barplot showing the number of alternative splicing events belonging to each of the main ASEs in Hs578-T cells with MS023 treatment for 5 days. **f**. Barplot showing the count of inverted-repeat (IR)-pairs in which the intron intersects with only the first Alu in the pair (1; white), or the only the second Alu in the pair (2; grey), or the two Alus in the pair (1/2; red). Source data

Having observed cytoplasmic dsRNA accumulation in response to type I PRMT inhibition, we then tried to identify the source of dsRNA and its relationship to inhibition of type I PRMTs. Unlike other epigenetic inhibitors, such as DNA methyltransferase inhibitors and lysine-specific demethylase inhibitors^33–35^ that induce endogenous retrovirus (ERV) expression, MS023 treatment did not significantly increase ERV expression (Extended Data Fig. 6a,b). It was recently reported that disruption of mRNA splicing through inhibition of the splicing regulatory pathway could trigger an antiviral response through the induction of misspliced RNAs. In particular, misspliced mRNAs with retained introns formed double-stranded structures that accumulated in the cytoplasm^36^. Given the broad effects of type I PRMTs on mRNA splicing^12,28^, we hypothesized that induction of dsRNA formation in response to MS023 treatment was due to direct deregulation of RNA splicing. To test this hypothesis, RNA-seq data were analyzed to identify disruptive alternative splicing events (ASEs) associated with MS023 treatment. A subset of predicted ASEs from splicing analysis was validated by qRT–PCR (Extended Data Fig. 6c,d). In total, 546 statistically significant differentially spliced events, including exon skipping, alternative splicing at the 3′ or 5′ site (A3SS/A5SS) and retained introns, distributed across 422 genes were identified following MS023 treatment (Fig. 6h). Importantly, retained introns are one of the major ASEs, suggesting that the dominant effect of type I PRMT inhibition on splicing is to induce intron retention.

**Extended Data Fig. 7:**
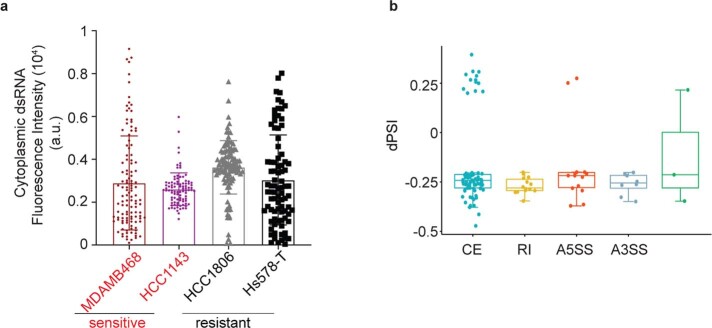
No correlation between IR-Alus induced dsRNA and basal IFN signature. **a**. Quantification of cytoplasmic dsRNA signal intensity across four TNBC cell lines (means±s.d., n = 3 independent biologically experiments of at least 85 cells per group analyzed). **b**. Distribution of dPSI for classes of alternative splicing events that correlates with MS023 sensitivity. Each dot represents a potential splicing event from n = 3 biological independent experiments. In the boxplots, the centre lines mark the median, the box limits indicate the 25th and 75th percentiles, and the whiskers extend to 1.5× the interquartile range from the 25th and 75th percentiles (unpaired two-tailed Student’s t-test). Source data

Recent studies have shown that intronic SINE elements, specifically inverted repeat (IR) Alu elements (IR-Alus), induced after treatment with DNA-hypomethylating agents, lead to immunogenic dsRNA^35–38^. We asked whether IR-Alus within retained introns could be the source of dsRNA induced by MS023 treatment. A search for sequences capable of forming IR-Alus within the MS023-associated retained introns revealed that 62% of the total 152 retained introns had sequences that intersected with IR-Alus, and about 50% of the retained introns had IR-Alu sequence pairs that were bidirectionally transcribed in both sense and antisense directions (Fig. 6i). However, only 10 IR-Alu sequences residing in 48 retained introns were identified in resistant Hs578-T cells, which is much less than in MDA-MB-468 cells (66 IR-Alus of 152 retained introns; Extended Data Fig. 6e,f). This low number of IR-Alu pairs might be insufficient to trigger dsRNA accumulation and subsequent antiviral-mediated IFN response. Immunopurification of dsRNA followed by qRT–PCR in MDA-MB-468 cells revealed enrichment for selected retained intron mRNAs with IR-Alus (*CD46* and *ASH1L*) but not two representative host mRNAs (*TUBB* and *ACTB*; Fig. 6j), confirming their contributions to the pool of dsRNA in response to type I PRMT inhibition in MS023-sensitive cell lines. Together, these results suggest that type I PRMT inhibition caused retained introns with IR-Alus, leading to dsRNA accumulation. Because we did not detect any preexisting difference of dsRNA accumulation or retained intron enrichment between MS023-sensitive and MS023-resistant cell lines, further investigations are required to identify the factors contributing to the basal IFN response gene expression signature and how these factors may prime TNBC cells to MS023 sensitivity (Extended Data Fig. 7a,b).

## Discussion

In this study, we took an unbiased epigenetic chemical screening approach and identified the type I PRMT inhibitor MS023 as capable of suppressing the proliferation of a subset of TNBC cells. Type I PRMTs have recently come into focus as promising targets due to their overexpression in many cancer types and the finding that their inhibition is tumor suppressive in many of these settings^12,17^. Specifically, in TNBC, our studies show that genetic and pharmacological inhibition of PRMT1, the main target among type I PRMTs, induces cell cycle arrest and apoptosis, leading to tumor suppression in a subset of TNBC. Moreover, preclinical models showed that type I PRMT inhibition suppressed tumor growth in human-derived models in vitro and in vivo.

Molecular-based prediction of drug response is a major goal of precision oncology. Given the complexity and heterogeneity of TNBC, the development of robust molecular predictors of drug response represents both an opportunity and a challenge. Although several factors, such as genomic deletion or epigenetic silencing of *MTAP* or *SRSF* mutation, have been linked to the responsiveness to type I PRMT inhibition, none of these molecular markers correlated with sensitivity to MS023 in the context of TNBC^17,28^. However, our gene expression data revealed that TNBC cells that are most sensitive to MS023 treatment have gene expression signatures enriched for IFN response and antiviral signaling. This correlation, which was confirmed in human-derived organoids, identifies an opportunity for the development of markers of innate immune response to stratify individuals most likely to benefit from type I PRMT inhibition. Our growth-inhibitory results align with reports of MS023 sensitivity in a subset of colorectal cancer organoids and human-derived glioblastoma stem cell lines^39,40^, which provide additional models to assess the dependency toward the innate immune response for sensitivity to PRMT1 inhibitors, such as MS023, but the underlying mechanisms were not investigated. Further study is needed to assess whether our observations in TNBC are generalizable to other human tumors.

Emerging evidence supports that some epigenetic-targeting molecules, such as inhibitors targeting DNA methyltransferase^34,35^, EZH2 (ref. ^41^), LSD1 (ref. ^33^), SETDB1 (ref. ^42^) and CARM1 (ref. ^43^), are capable of inducing robust antitumor immune responses. These effects are the result of induced ‘viral mimicry effects’, increased tumor antigen expression/presentation, T cell activation, a remodeled tumor microenvironment or combinations thereof. Here, we discovered a new link between type I PRMT inhibition and innate antitumor immunity. Pharmacological inhibition of type I PRMT amplifies preexisting IFN responses by inducing cytosolic dsRNA accumulation in sensitive TNBC lines. Unlike many of the other epigenetic inhibitors that induce ERV expression, MS023 treatment triggers dsRNA accumulation from intron-retained RNAs. While PRMT enzymes are often classified as epigenetic targets based on their histone substrates, PRMTs have a much broader set of targets, especially RNA-binding proteins involved in mRNA splicing. Given these broad roles of type I PRMT on RNA splicing, our discovery is consistent with the recent report showing that spliceosome-targeted therapies induce intron-retained transcripts and dsRNA formation^36^.

In summary, our results show that type I PRMT inhibition results in potent antitumor activity associated with increased IFN response and dsRNA accumulation derived from misspliced RNAs. Notably, the innate immune effects of type I PRMT inhibition are restricted to TNBC cells with preexisting elevated IFN response gene expression signatures, suggesting that MS023 treatment may push TNBC cells that are already stressed over a threshold to induce cell death. The identification of biomarkers of type I inhibition sensitivity provides important insights into the targeted development of this class of therapy in TNBC and other cancers. Furthermore, given the general role of type I PRMT inhibitors in stimulating dsRNA and IFN response signals, targeting type I PRMTs combined with immune checkpoint inhibition may offer potential new opportunities for cancer immunotherapy.

## Methods

### Cell culture

TNBC cell lines were obtained from ATCC and cultured according to ATCC’s recommendations with Dulbecco’s modified Eagle’s medium (DMEM; Gibco) or RPMI 1640 (Life Technologies, 11965) with 10% FBS (Merck, F12103) and 1% penicillin/streptomycin (Gibco, 15140122). Cells were maintained mycoplasma free by using a MycoAlert mycoplasma detection kit (Lonza) and were passaged no more than 25 times.

### Epigenetic chemical screen

All compounds were purchased from Cayman Chemical, Millipore-Sigma or MedChemExpress. The detailed resources for each compound are listed in the Supplementary Information. Chemical purity was validated at the SGC at more than 99%. TNBC cell lines were plated at 500 cells per well on 384-well plates. After cells adhered to the plates, compounds were dissolved in DMSO and added to achieve a final concentration of 5 μM. Each plate contained four replicates for each compound and DMSO as a control. Cells were then placed in an IncuCyte ZOOM live-cell analysis system (Essen492 Biosciences) for 6 d. Confluency was imaged with a ×10 objective in phase-contrast mode and analyzed using Incucyte 2016 integrated software, according to manufacturer’s instructions. Data were normalized to the DMSO control wells, and the log_2_ average confluency values are presented.

### Western blotting

Cells and tumor tissues were collected and lysed as described before^44^. Briefly, 20–100 μg of protein was boiled at 95 °C for 5 min, loaded onto NuPAGE 4–12% Bis-Tris protein gels (Invitrogen) and transferred to nitrocellulose membranes (Bio-Rad) using the Mini-PROTEAN Tetra electrophoresis system (Bio-Rad). Primary antibodies used for membrane staining were anti-PRMT1 (Millipore, 7404; 1:1,000), anti-ADMA (Cell Signaling, 13522S; 1:1,000), anti-GAPDH (Santa Cruz Biotechnology, sc-32233; 1:5,000), anti-pH2A.X (Cell Signaling, 9718S; 1:1,000), anti-β-actin (Santa Cruz Biotechnology, sc-47778; 1:3,000) and anti-dsRNA (J2) (Scicons, 10010200; 1:5,000). Images were collected on an Odyssey scanner (LiCor) or Amershan ImageQuant 80 and analyzed with ImageQuant TL (v8.2.0) and ImageJ (v1.53a).

### Cell cycle assay

Cells were plated in six-well plates and treated with 1 μM MS023 or DMSO as a control. An allophycocyanin BrdU flow kit (BD Pharmingen) was used for the following analysis. Cells were incubated for 6 h with 10 μM BrdU and fixed and permeabilized with BD Cytofix/Cytoperm buffer. Immunofluorescent cell staining with fluorochrome-conjugated anti-BrdU and 7-aminoactinomycin D was performed following the manufacturer’s instructions and analyzed using a BD FACScan flow cytometer. The number of live cells in each stage was determined (FlowJo software, version 9.3.1).

### Cell apoptosis assay

Cells were plated at 3,000 cells per well in 96-well plates with DMSO or MS023 for 5 d of treatment. Caspase-3/caspase-7 green apoptosis assay reagent (Sartorius, 4440) was added to the cells and imaged and analyzed with an Incucyte ZOOM 2FLR (v.1.00) system according to manufacturer’s instruction.

### Cell viability assay

Cells were seeded and treated with prescribed conditions in 384-well plates accordingly. At the endpoint, 25 µl of Promega CellTiter-Glo luminescent cell viability assay reagent (Promega, G7572) was added to each well. Plates were mixed gently for 2 min on an orbital shaker and incubated at room temperature for 20 min protected from light. The luminescent signal intensity was subsequently read on a CLARIOstar plate reader (BMG LABTECH).

### Gene set enrichment analysis

Compound activity and cell viability data were collected and processed using an IncuCyte ZOOM live-cell analysis system (Essen492 Biosciences). Data were normalized to the DMSO control and were analyzed using R (v3.5.1) with Bioconductor (v.3.14). Cell half-maximal inhibitory concentration (IC_50_) and AAC^45^ values were analyzed using PharmacoGx R package (v. 2.6.0). We obtained the transcriptome data of the 17 cell lines through RNA-seq and processed the data with the Kallisto pipeline^3^. For all 17 cell lines, genes were ranked based on Pearson correlation coefficients between the measured compound activity (IC_50_) and individual gene expression level. Hallmarks gene sets were downloaded from MsigDB (https://www.gsea-msigdb.org/gsea/msigdb/)^46^. The piano R package was used to generate the GSEA results, and the fgsea R package was used to create pathway enrichment plots^47,39^.

### Proteomics analysis

Princess Margaret Cancer Centre protein expression data were downloaded from ref. ^27^, and MD Anderson protein expression data were downloaded from ref. ^48^. The relative correlations between individual protein expression level and compound effectiveness (IC_50_) were analyzed by Pearson correlation coefficients using GraphPad Prism 8.0.

### Splicing data analysis

ASEs were identified for the human genome (hg38) using the vast-tools pipeline^49,50^ with |dPSI | ≥ 0.2 and MV | dPSI_at_95 | ≥ 0.05 for significance, and additional non-default parameters for vast-tools diff module include -S 3, -e 10, -m 0.01. ASEs were further categorized into functional classifications according to their predicted impact on the open reading frame (ORF) as ‘neutral’ for events that generate known functional isoforms or that do not alter the protein sequence (for example, an alternative exon), ‘protective’ for events that reduce the occurrence of deleterious nucleotide sequences and therefore generate a functional protein (for example, removal of an intron/exon containing a premature stop codon) and ‘deleterious’, which denotes events that increase the frequency of disruptive sequences in the ORF (for example, inclusion of introns/exons containing premature stop codons or removal of essential exons for protein function)^51^. All postprocessing data analysis and figure generation steps were conducted using custom Python 3.7 scripts, which are available upon request.

### Lentiviral mRNA targets

*PRMT1*-targeting shRNA vector was a kind gift from the laboratory of C. Strahdee, and the sequences are listed in Supplementary Table 4. Target and packaging plasmid were cotransfected into HEK293 cells to produce lentivirus. Lentivirus was transfected into cell lines along with polybrene (8 μg ml^–1^). Fresh medium was replaced after 16 h, and puromycin selection (0.2–0.6 μg ml^–1^) began after 48 h. After 72 h, the selection was complete, and stable transduced cells were generated.

### dsRNA immunoprecipitation

MDA-MB-468 cells were seeded at 1.0 × 10^6^ in 10-cm dishes. After cells adhered for 24 h, 1 μM MS023 or DMSO was added to treat cells for 5 d. Cells were then trypsinized and washed with ice-old PBS. Cells were centrifuged (4 °C,180*g*, 5 min), and the supernatant was discarded. Cell pellets were lysed in 1 ml of RIP buffer (25 mM HEPES pH 7.2, 150 mM NaCl, 5 mM MgCl_2_, 0.1% Igepal CA-630 and 1 U µl^–1^ Rnasin Plus) for 5 min on ice. After cell lysis, tubes were centrifuged, and the supernatant was transferred into a new Eppendorf tube; 10% of the supernatant was used for total RNA extraction with Trizol, and the rest was used for dsRNA immunoprecipitation.

Protein A Dynabeads were prepared by using NT-2 buffer (50 mM Tris-Cl, pH 7.4, 150 mM NaCl, 1 mM MgCl_2_ and 0.1% Igepal CA-630) for washing and resuspending. Five micrograms of anti-dsRNA (J2) (monoclonal) was added to 100 µl of beads and incubated at 4 °C overnight. The next day, cell lysate was co-incubated with 100 µl of J2-bound Protein A Dynabeads at 4 °C for 3 h. Beads were washed with NT-2 buffer three times, followed by washing with high-salt wash buffer (50 mM Tris-Cl, pH 7.4, 300 mM NaCl, 1 mM MgCl_2_, 0.5% Igepal CA-630 and 0.1% SDS) three times. Trizol was used to collect J2-bound dsRNA from beads. Chloroform was added at a 1:5 ratio, and RNA was cleaned using an RNA Clean and Concentrator kit (Zymo).

### Quantitative real-time PCR

An iScript gDNA Clear cDNA Synthesis kit (Bio-Rad) was used for synthesizing cDNA. According to the manufacturer’s protocol, 1 µg of total RNA or dsRNA was used to generate a 20-µl reaction mixture. A 10× dilution was then made by adding 180 µl of double-distilled water, and 2 µl of synthesized cDNA was used per reaction. During qRT–PCR, cDNA template and PowerUp SYBR Green master mix (Applied Biosystems) were added into white PCR reaction tubes and placed in a CFX Maestro (Bio-Rad). All experiments were performed in at least three biological replicates. Primers were designed to measure fully spliced transcripts and intron-containing transcripts, and all primer sequences are provided in Supplementary Table 3. For data analysis, threshold cycle (*C*_t_) values obtained during qRT–PCR were used to calculate the ratio of intron-containing transcripts to fully spliced transcripts. Data were collected on a Bio-Rad CFX96 touch and analyzed by Bio-Rad CFX Manager (v3.1.1517.0823).

### Transposable elements analysis

FASTQ files were aligned to the human genome (Grch38) with Bowtie2 (v2.2.5). After converting the .sam files to .bam files using samtools (v1.10), we used the Repenrich2_subset function to generate discrete files for uniquely and multimapped reads. These reads were further analyzed to estimate the expression level of repetitive elements by the Repenrich2 function. DESeq2 (v1.34) was used to analyze the differential expression of estimated repeats counts.

### dsRNA and dsDNA immunofluorescence staining

Cells were grown on poly-l-ornithine-pretreated coverslips placed in 12-well plates. Knockout cells were induced by 1 μg ml^–1^ doxycycline for 3 d, and compound-treated cells were treated with 1 μM MS023 or DMSO for 5 d. After treatment, cells were washed in PBS and fixed in 4% formaldehyde for 15 min at room temperature. Cells were then washed three times with PBS, permeabilized in 0.5% (vol/vol) Triton X-100 for 10 min and blocked in 5% bovine serum albumin in PBS for 1 h at room temperature. Primary antibody was added (anti-dsRNA J2 (Scicons, 10010200) diluted 1:500 and anti-dsDNA (Abcam, ab27156) diluted 1:1,000) and incubated at 4 °C overnight. Secondary anti-mouse IgG Alexa 647 (Cell Signaling Technology, 4410S) was diluted 1:1,000 and incubated for 1 h in a black container at room temperature. Coverslips were washed with PBS, and DAPI-containing mountant (Invitrogen ProLong Gold Antifade Mountant, P36930) was used. Images were collected on a A1 HD25 Single-Photon confocal microscope and analyzed by NIS-Elements (v5.21.03).

### Organoid assay

BXTO.64, DCBXTO.58 and DCBXTO.132 organoids were derived from PDX tumors. DCBPTO.66 organoids were derived from human tumors. Human tumors were collected with informed participant consent according to University Health Network-approved Research Ethics Board protocols (14-8358). Tumor tissue was minced and digested in 5–10 ml of Advanced DMEM containing 1× GlutaMAX, 10 mM HEPES, 1× antibiotic–antimycotic (AdDF+++) and 250–500 μg ml^–1^ Liberase TH for 45 min at 37 °C with gentle rocking. Tissue was filtered over a 100-μm cell strainer and pelleted by centrifugation at 400*g* for 10 min at 4 °C. Pellets were washed once with AdDF+++, repelleted and treated with Red Cell Lysis Buffer Hybri-Max for 5 min on ice before cell counting. Organoids were cultured in medium as previously described^30^. PDX-derived organoids were regularly evaluated for human and mouse cell content by flow cytometry to ensure purity. Short tandem repeat analysis was used to confirm that the organoids matched their tumor of origin, and mycoplasma testing was performed as a quality control step.

For drug assays, organoids were dissociated into single cells, and 2,000 cells were plated per well in duplicate in 25 µl of basement membrane extracts (BME) in 48-well plates. Once the BME had solidified, the organoid/BME domes were overlaid with 475 µl of medium with or without drug. Fresh medium and drug were applied every 5 d. A well containing BME only (no cells) was included as a medium-only control. The cells were cultured for 12–21 d (depending on the growth rate of the model) until the untreated controls had formed organoids of >50 mm in diameter. Medium was removed, and the organoids were incubated with 1× PrestoBlue HS Reagent (Thermo Fisher) in Breast Organoid Media^30^ overnight at 37 °C. The following day, aliquots of the medium supernatant were transferred to a 384-well plate, and fluorescence readings were taken at 560/590-nm excitation/emission wavelength using a CLARIOstar Plus microplate reader. The medium-only control was used for background correction, and cell viability for each organoid model was normalized to its respective no-drug control. Three independent assays were done per organoid model.

### Mouse studies

All animal experiments were reviewed and approved by the Animal Care Committee at the University Health Network in Toronto. For in vivo dosing experiments, 7 × 10^6^ MDA-MB-468 cells were injected into the mammary fat pad of severe combined immunodeficient mice following standard procedures. MS023 was prepared at 5% N-Methyl-2-pyrrolidone (NMP), 20% captisol (wt/vol), 20% PEG-400 and 55% normal saline and administered to the mice by intraperitoneal injection. The MS023-sensitive cell line (MDA-MB-468) used in this experiment has a tendency to ulcerate through the skin when it reaches a modest size, which precludes continuation of experiments to larger tumor volumes due to humane endpoints. Thus, we allowed tumors to grow to a detectable size, as assessed by palpation (~2 mm in diameter), and started to treat with 60 mg kg^–1^ MS023 daily for a total of 5 weeks. Animals were randomized to treatment via random number generation/assignment. Body weights and tumor growth were measured once a week over the course of treatment until the endpoint was reached.

### Reporting Summary

Further information on research design is available in the Nature Research Reporting Summary linked to this article.

## Online content

Any methods, additional references, Nature Research reporting summaries, source data, extended data, supplementary information, acknowledgements, peer review information; details of author contributions and competing interests; and statements of data and code availability are available at 10.1038/s41589-022-01024-4.

## Supplementary information

Supplementary Information Supplementary Tables 1–5 and Fig. 1.

Reporting Summary

## Data Availability

Gene dependency data are available at depmap bioportal (https://depmap.org/portal/). RNA/protein expression data for TNBC cell lines are available in the datasets GSE73526 and GSE74702. RNA expression data for human breast cancer samples are available at TCGA (https://portal.gdc.cancer.gov/) and METABRIC (https://www.cbioportal.org/) and for the cell lines at GSE73526 and GSE74702. RNA expression data for PDX models are available upon request to protect participant privacy. The gene sets used for GSEA analysis are available at the molecular signatures database (https://www.gsea-msigdb.org/gsea/msigdb/). The RNA-seq data generated in this article are available from http://neellab.github.io/bfg/. Other data are available from the corresponding authors upon reasonable request. Source data are provided with this paper.

## Source data

Source Data Fig. 1 Statistical source data.
Source Data Fig. 2 Statistical source data.
Source Data Fig. 2 Unprocessed western blots.
Source Data Fig. 3 Statistical source data.
Source Data Fig. 4 Statistical source data.
Source Data Fig. 4 Unprocessed western blots.
Source Data Fig. 5 Statistical source data.
Source Data Fig. 6 Statistical source data.
Source Data Extended Data Fig. 1 Statistical source data.
Source Data Extended Data Fig. 2 Statistical source data.
Source Data Extended Data Fig. 2 Unprocessed western blots.
Source Data Extended Data Fig. 3 Statistical source data.
Source Data Extended Data Fig. 4 Statistical source data.
Source Data Extended Data Fig. 4 Unprocessed western blots.
Source Data Extended Data Fig. 5 Statistical source data.
Source Data Extended Data Fig. 6 Statistical source data.
Source Data Extended Data Fig. 7 Statistical source data.

## Code availability

Code to reproduce the RNA-seq bioinformatics analyses and the related data are available at https://github.com/bhklab/TNBC_PRMT1i, and code to reproduce the RNA splicing analyses is available at https://gist.github.com/Fciamponi/ef8aae813575a4947c1f9cb87a1c7330. Code to reproduce the ERV analysis is available at https://github.com/WuEpiLab/TNBC_MS023_ERV.
