# Supplementary material for: PRMT inhibition induces a viral mimicry response in triple-negative breast cancer
Source: Nat Chem Biol. 2022 May 16;18(8):821–30. doi: 10.1038/s41589-022-01024-4 (PMC9337992; doi:10.1038/s41589-022-01024-4)
Supplement: Supplementary file 1 — Supplementary Tables 1–5 and Fig. 1. [file 41589_2022_1024_MOESM1_ESM.pdf]

nature portfolio

https://doi.org/10.1038/s41589-022-01024-4

Supplementary information

PRMT inhibition induces a viral mimicry response in triple-negative breast cancer

In the format provided by the authors and unedited

Protein Family	Target	Probe	PubMed ID	Source
Methyltransferase	SUV20H1/H2	A-196	28114273	Cayman Chemical
Methyltransferase	EHMT2, EHMT1	A-366	24900801	MilliporeSigma
Methyltransferase	EED	A-395	28135237	MilliporeSigma
Methyltransferase	SMYD2	BAY-598	27075367	MilliporeSigma
Bromodomain	BAZ2A/2B	BAZ2-ICR	25719566	MilliporeSigma
Bromodomain	BRD9, BRD7	BI9564	26914985	MilliporeSigma
Methyltransferase	EZH2	GSK126	23051747	MedChemExpress
Bromodomain	BAZ2A/2B	GSK2801	25799074	MilliporeSigma
Methyltransferase	EZH2	GSK343	24900432	MilliporeSigma
PAD	PAD-4	GSK484	25622091	MilliporeSigma
Methyltransferase	PRMT5	GSK591	26985292	MilliporeSigma
DEHYDR	Dehydrogenasel	GSK864	26436839	MilliporeSigma
KDM	KDM6B, KDM6A	GSKJ4	22842901	MilliporeSigma
KDM	KDM1A	GSKLSD1	26175415	MilliporeSigma
Bromodomain	BRD9	I-BRD9	25856009	MilliporeSigma
Bromodomain	CREBBP P300	I-CBP112	26552700	MilliporeSigma
PHD	pan-2-OG	IXO2	28656004	MilliporeSigma
Bromodomain	BRD2-4	JQ-1	20871596	Cayman Chemical
Bromodomain	KAT2A, KAT2B	L-MOSES	27966810	Bio-Techne Ltd.
Bromodomain	BRD9, BRD7	LP99	25864491	Bio-Techne Ltd.
Methyltransferase	TYPE1 PRMT	MS023	26598975	MilliporeSigma
Methyltransferase	PRMT4, PRMT6	MS049	27584694	MilliporeSigma
Bromodomain	BRPF1-3	NI-57	28714688	MilliporeSigma
Bromodomain	BRPF1-3	OF-1	21804994	Bio-Techne Ltd.
Bromodomain	BRD2-4	PFI-1	23095041	MilliporeSigma
Methyltransferase	SETD7	PFI-2	25136132	MilliporeSigma
Bromodomain	SMARCA2,4	PFI-3	26139243	MedChemExpress
Bromodomain	BRPF1B	PFI-4	28849908	Bio-Techne Ltd.
Methyltransferase	DOT1L	SGC0946	23250418	SGC
Methyltransferase	PRMT3	SGC707	25728001	SGC
Bromodomain	CREBBP P300	SGC-CBP30	24946055	Cayman Chemical
Methyltransferase	PRMT4	TP-064	29719619	MilliporeSigma
Bromodomain	BRD9, BRD7	TP-472	34771678	MilliporeSigma
Methyltransferase	EHMT2, EHMT1	UNC0638	21743462	MilliporeSigma
Kme	L3MBTL3	UNC1215	23292653	Bio-Techne Ltd.
Methyltransferase	EZH2	UNC1999	23614352	MilliporeSigma
KDM	KDM6B, KDM6A	GSKJ5	22842901	Cayman Chemical
Bromodomain	BRD2-4	JQ1N	20871596	MilliporeSigma
Methyltransferase	TYPE1 PRMT	MS094	26598975	MilliporeSigma
Methyltransferase	PRMT5	SGC2096	26985292	SGC
Methyltransferase	EZH2	UNC2400	23614352	Bio-Techne Ltd.,

Supplementary Table 1: List of small molecule inhibitors in the epigenetic probe library used for screening TNBCs, alongside their protein family, specific target, and publication details.

Type I PRMTs	Kd (nM)			
	**MS023**	SGC707	MS049	TP-064
PRMT1	39			
PRMT3	135	31		
PRMT4	93		44	<10
PRMT6	4		63	
PRMT8	5			

Supplementary Table 2: *In Vitro* Kd value for indicated chemical probes.

Primer list		
	Forward	Reverse
ASH1L-1	AAGAGTGTCAAGTGGGTGGAC	ATGGGGTTTCGTCATGTTGG
CD46	ATTACAGGCACCCACCACTATG	ATCACTTGAGGTCAGGAGTTCG
OAS1	GATCTCAGAAATACCCCAGCCA	AGCTACCTCGGAAGCACCTT
MX1	CTCCGACACGAGTTCCACAA	GGCTCTTCCAGTGCCTTGAT
MAVS	CTGCTCACCAAGGTGTCT	CTGAGCTGCCTCCAGTG
MDA5	GGGGCATGGAGAATAACTCA	AGCTGACACTTCCTTCTGCC
RIG-I	ATATCCGGAAGACCCTGGAC	GAGAAAAAGTGTGGCAGCCT
IFNalpa	AATGACAGAATTCATGAAAGCGT	GGAGGTTGTCAGAGCAGA
IFNbeta	GCCATCAGTCACTTAAACAGC	GAAACTGAAGATCTCCTAGCCT
TLR3	TGGTTGGGCCACCTAGAAGTA	TCTCCATTCCTGGCCTGTG
STING	AGCATTACAACAACCTGCTACG	GTTGGGGTCAGCCATACTCAG
cGAS	TAACCCTGGCTTTGGAATCAAAA	TGGGTACAAGGTAAAATGGCTTT
GAPDH	GGGGTCATTGATGGCAACAATA	AAGGTGAAGGTCGGAGTCAAC
HERV-E	GGTGTCACTACTCAATACAC	GCAGCCTAGGTCTCTGG
HERV-F	CCTCCAGTCACAACAACTC	TATTGAAGAAGGCGGCTGG
actin	CACCATTGGCAATGAGCGGTTC	AGGTCTTTGCGGATGTCCACGT
a-TUBULIN	TCTGTTAGTGGGAGATCCTT	TGGGTTCCAAGTCTACAAAC

Supplementary Table 3: Primers for qRT-PCR.

shRNA	Target sequence
shPRMT1-3	GTGTTCCAGTATCTCTGATTA
shPRMT1-5	GCAAGTGAAGCGGAATGACTA
shPRMT1-250	AAGCCCAACGCTGAGGACATGACAT

Supplementary Table 4: Target sequences of shRNA-PRMT1.

Organoid Name	Organoid	Gender	Age Range	Histology	Tumour Source	Treatment History
BXTO.64	PDXO	Female	60-69	Invasive ductal carcinoma	Metastatic site (unknown)	DOXORUBICIN, CYCLOPHOSPHAMIDE, PACLITAXEL
DCBXTO.58	PDXO	Female	30-39	Invasive ductal carcinoma	Metastatic site (liver)	FLUOROURACIL, EPIRUBICIN, CYCLOPHOSPHAMIDE, DOCETAXEL, LETROZOLE, PALBOCYCLIB, FULVESTRANT, MOLIBRESIB
DCBXTO.132	PDXO	Female	40-49	Invasive ductal carcinoma	Metastatic site (chest wall)	PACLITAXEL, TRASTUZUMAB, PERTUZUMAB, ZOLEDRONIC ACID
DCBPTO.66	PDO	Female	60-69	Invasive ductal carcinoma	Metastatic site (chest wall)	DOXORUBICIN, CYCLOPHOSPHAMIDE, PACLITAXEL

Supplementary Table 5: Sample characteristics. Abbreviations: PDXO: patient derived xenograft organoid; PDO: patient derived organoid.

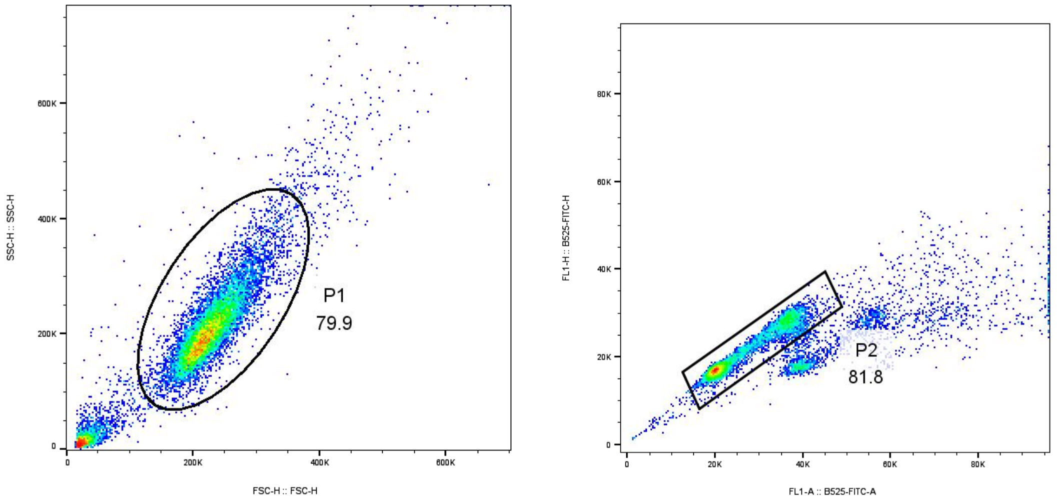

Supplementary Fig.1. Representative plots for gating strategy of cell cycle analysis.
