# Supplementary material for: PRMT inhibition induces a viral mimicry response in triple-negative breast cancer
Source: Nat Chem Biol. 2022 May 16;18(8):821–30. doi: 10.1038/s41589-022-01024-4 (PMC9337992; doi:10.1038/s41589-022-01024-4)
Supplement: Supplementary file 2 — Reporting Summary [file 41589_2022_1024_MOESM2_ESM.pdf]

nature research

Corresponding author(s): Cheryl Arrowsmith, Qin Wu, Mathieu Lupien

Last updated by author(s): Jun 21, 2021

# Reporting Summary

Nature Research wishes to improve the reproducibility of the work that we publish. This form provides structure for consistency and transparency in reporting. For further information on Nature Research policies, see Authors & Referees and the Editorial Policy Checklist.

## Statistics

For all statistical analyses, confirm that the following items are present in the figure legend, table legend, main text, or Methods section.

| n/a | Confirmed | |
|---|---|---|
| ☐ | ☒ | The exact sample size (*n*) for each experimental group/condition, given as a discrete number and unit of measurement |
| ☐ | ☒ | A statement on whether measurements were taken from distinct samples or whether the same sample was measured repeatedly |
| ☐ | ☒ | The statistical test(s) used AND whether they are one- or two-sided<br>*Only common tests should be described solely by name; describe more complex techniques in the Methods section.* |
| ☐ | ☐ | A description of all covariates tested |
| ☐ | ☒ | A description of any assumptions or corrections, such as tests of normality and adjustment for multiple comparisons |
| ☐ | ☐ | A full description of the statistical parameters including central tendency (e.g. means) or other basic estimates (e.g. regression coefficient) AND variation (e.g. standard deviation) or associated estimates of uncertainty (e.g. confidence intervals) |
| ☒ | ☐ | For null hypothesis testing, the test statistic (e.g. *F*, *t*, *r*) with confidence intervals, effect sizes, degrees of freedom and *P* value noted<br>*Give P values as exact values whenever suitable.* |
| ☒ | ☐ | For Bayesian analysis, information on the choice of priors and Markov chain Monte Carlo settings |
| ☒ | ☐ | For hierarchical and complex designs, identification of the appropriate level for tests and full reporting of outcomes |
| ☐ | ☒ | Estimates of effect sizes (e.g. Cohen's *d*, Pearson's *r*), indicating how they were calculated |

*Our web collection on statistics for biologists contains articles on many of the points above.*

## Software and code

Policy information about availability of computer code

| | |
|---|---|
| Data collection | Incucyte Zoom 2016A<br>Odyssey scanner (LiCor) |
| Data analysis | Incucyte ZOOM 2016A<br>Prism Version 7.0c<br>R (V.3.5.1)<br>Bioconductor (v.3.7)<br>FlowJo software (version 9.3.1)<br>ExcelMacro Report Generator Version 3.0.3<br>IncucyteDRC R package |

For manuscripts utilizing custom algorithms or software that are central to the research but not yet described in published literature, software must be made available to editors/reviewers. We strongly encourage code deposition in a community repository (e.g. GitHub). See the Nature Research guidelines for submitting code & software for further information.

## Data

Policy information about availability of data

All manuscripts must include a data availability statement. This statement should provide the following information, where applicable:

- Accession codes, unique identifiers, or web links for publicly available datasets
- A list of figures that have associated raw data
- A description of any restrictions on data availability

The data supporting the findings of this study are available within the paper and its supplementary information files and are available from the corresponding authors. Code to reproduce the bioinformatics analyses and their related data is available at https://github.com/bhklab/TNBC_BAY-876.

# Field-specific reporting

Please select the one below that is the best fit for your research. If you are not sure, read the appropriate sections before making your selection.

☒ Life sciences ☐ Behavioural & social sciences ☐ Ecological, evolutionary & environmental sciences

For a reference copy of the document with all sections, see nature.com/documents/nr-reporting-summary-flat.pdf

# Life sciences study design

All studies must disclose on these points even when the disclosure is negative.

| | |
|---|---|
| Sample size | Sample sizes of 3 biological replicates and two or three individual experiments are sufficient to determine significant differences for changes in the analyzed biological products. |
| Data exclusions | No data has been excluded from this study |
| Replication | All findings using the inhibitors to alter growth of cell lines, organoids and explants were reproducible over several experiment. |
| Randomization | Randomization was not relevant to this study since no mice work is included in this work. |
| Blinding | Blinding was not relevant to this study as all biological samples were equally analyzed. |

# Reporting for specific materials, systems and methods

We require information from authors about some types of materials, experimental systems and methods used in many studies. Here, indicate whether each material, system or method listed is relevant to your study. If you are not sure if a list item applies to your research, read the appropriate section before selecting a response.

### Materials & experimental systems

| n/a | Involved in the study | |
|---|---|---|
| ☐ | ☒ | Antibodies |
| ☐ | ☒ | Eukaryotic cell lines |
| ☒ | ☐ | Palaeontology |
| ☐ | ☒ | Animals and other organisms |
| ☒ | ☐ | Human research participants |
| ☒ | ☐ | Clinical data |

### Methods

| n/a | Involved in the study | |
|---|---|---|
| ☒ | ☐ | ChIP-seq |
| ☒ | ☐ | Flow cytometry |
| ☒ | ☐ | MRI-based neuroimaging |

## Antibodies

| | |
|---|---|
| Antibodies used | anti-PRMT1 (Millipore) (7404)<br>anti-ADMA (EPR) (Cell Signaling) (13522S)<br>anti-GAPDH (Santa Cruz Biotechnology)(sc-32233)<br>goat-anti rabbit (IR800 conjugated, LiCor no. 926-32211)<br>donkey anti-mouse (IR 680, LiCor no. 926-68072)<br>anti-pH2A.X (Cell Signaling) (13522S)<br>anti-actin (santa Cruz Biotechnology) ( sc-47778)<br>anti-dsRNA (J2) (Scucibs) (10010200) |
| Validation | see websites of the manufacturers |

## Eukaryotic cell lines

Policy information about cell lines

| | |
|---|---|
| Cell line source(s) | ATCC |
| Authentication | none were authenticated |
| Mycoplasma contamination | All cell lines tested negative for mycoplasma |
| Commonly misidentified lines (See ICLAC register) | *Name any commonly misidentified cell lines used in the study and provide a rationale for their use.* |

## Animals and other organisms

Policy information about studies involving animals; ARRIVE guidelines recommended for reporting animal research

| | |
|---|---|
| Laboratory animals | SCID mice |
| Wild animals | *Provide details on animals observed in or captured in the field; report species, sex and age where possible. Describe how animals were caught and transported and what happened to captive animals after the study (if killed, explain why and describe method; if released, say where and when) OR state that the study did not involve wild animals.* |
| Field-collected samples | *For laboratory work with field-collected samples, describe all relevant parameters such as housing, maintenance, temperature, photoperiod and end-of-experiment protocol OR state that the study did not involve samples collected from the field.* |
| Ethics oversight | All animal experiments were reviewed and approved by the Animal Care Committee at the University Health Network in Toronto. |

Note that full information on the approval of the study protocol must also be provided in the manuscript.
