# Supplementary figures and images for: PRMT inhibition induces a viral mimicry response in triple-negative breast cancer
Source: Nat Chem Biol. 2022 May 16;18(8):821–30. doi: 10.1038/s41589-022-01024-4 (PMC9337992; doi:10.1038/s41589-022-01024-4)

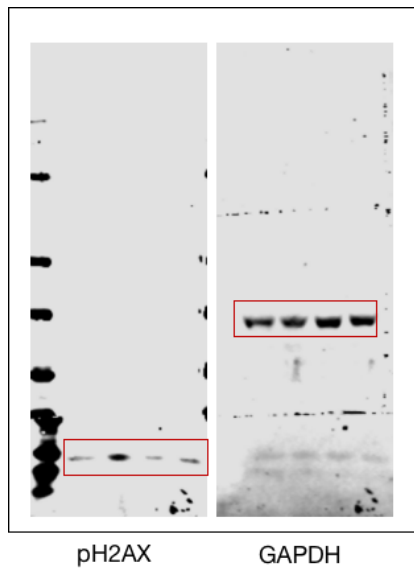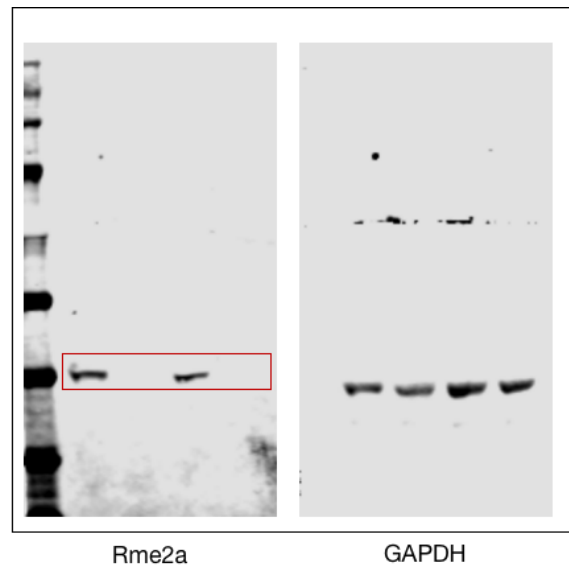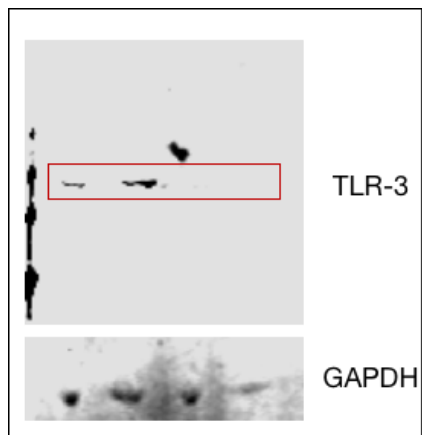

Source images for western blots shown in Fig. 4d

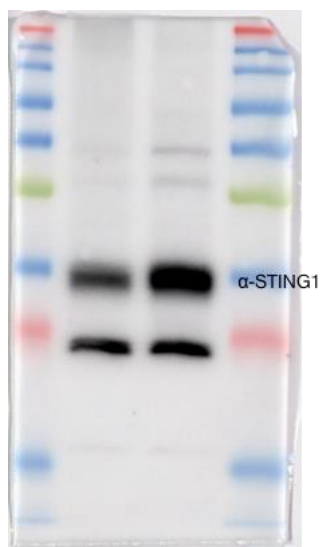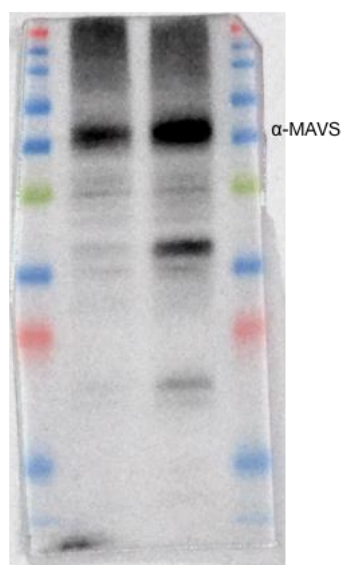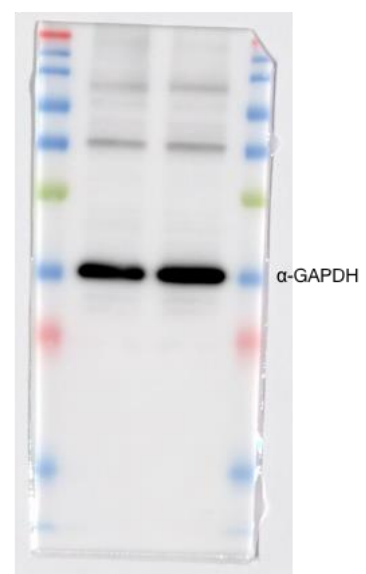

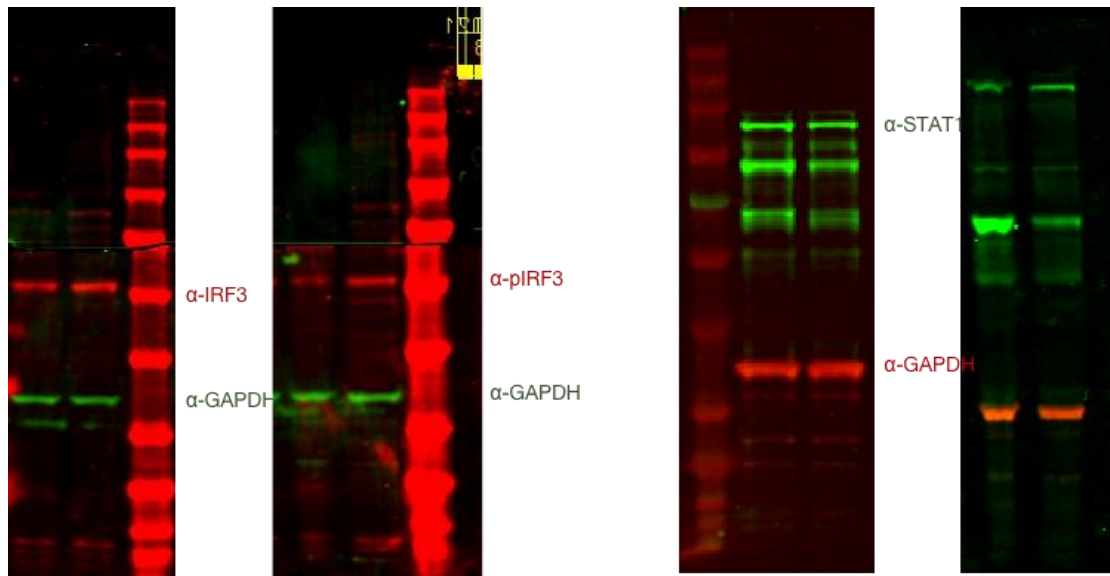

Source images for western blots shown in Fig. 4g.

Supplement: Source Data Fig. 4 — Unprocessed western blots. [file 41589_2022_1024_MOESM8_ESM.pdf]

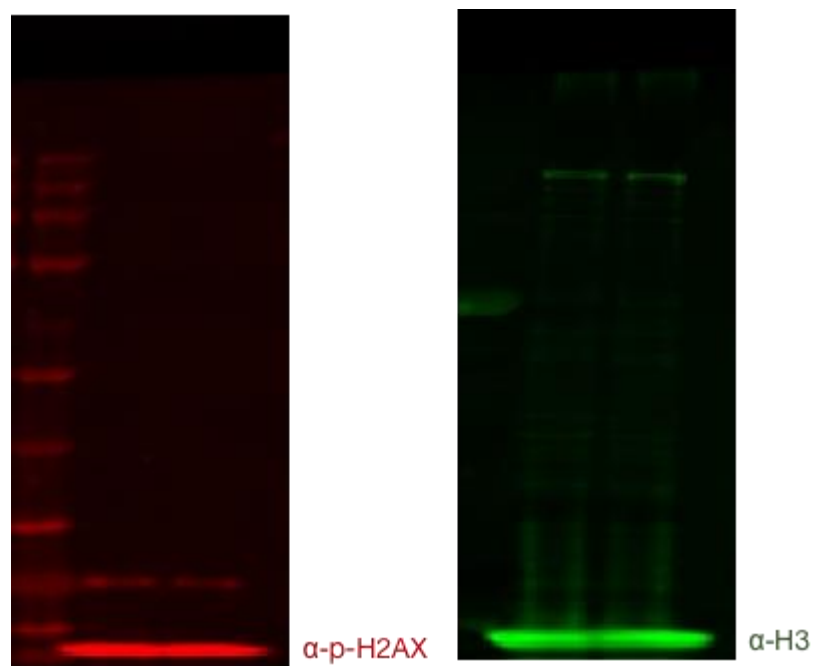

Source images for western blots shown in Extended Data Fig. 4h.

Supplement: Source Data Extended Data Fig. 4 — Unprocessed western blots. [file 41589_2022_1024_MOESM16_ESM.pdf]
