# Supplementary material for: PRMT inhibition induces a viral mimicry response in triple-negative breast cancer
Source: Nat Chem Biol. 2022 May 16;18(8):821–30. doi: 10.1038/s41589-022-01024-4 (PMC9337992; doi:10.1038/s41589-022-01024-4)
Supplement: Source Data Extended Data Fig. 2 — Unprocessed western blots. [file 41589_2022_1024_MOESM13_ESM.pdf]

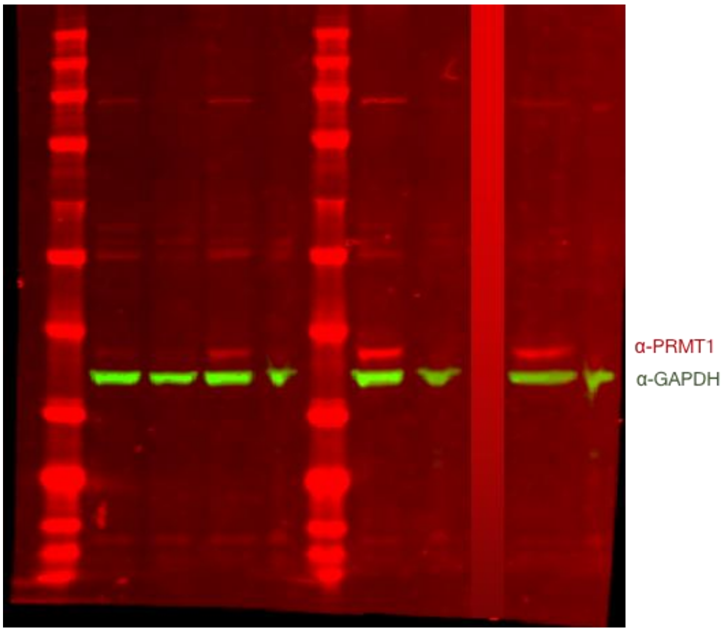

Source images for western blots shown in Extended Data Fig. 2c.

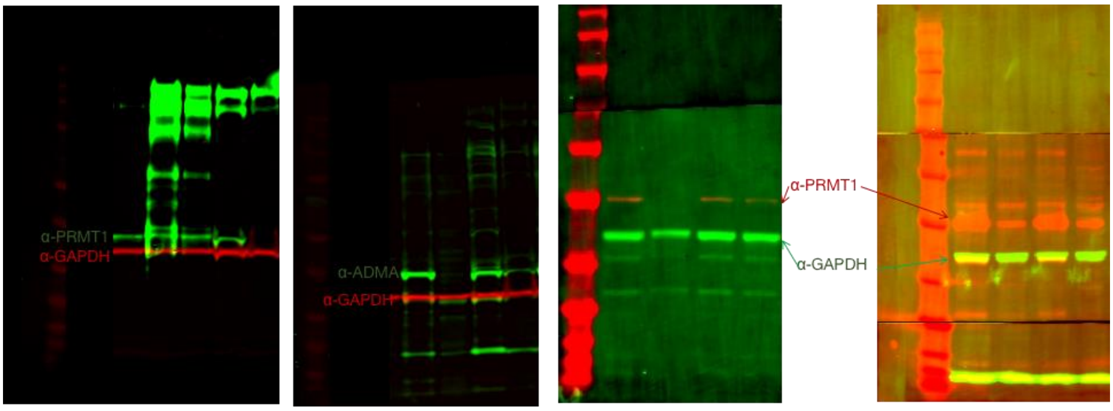

Source images for western blots shown in Extended Data Fig. 2f.
